# Spontaneous oxycodone withdrawal disrupts sleep, diurnal, and electrophysiological dynamics in rats

**DOI:** 10.1371/journal.pone.0312794

**Published:** 2025-01-17

**Authors:** Michael Gulledge, William A. Carlezon, R. Kathryn McHugh, Elizabeth A. Kinard, Michael J. Prerau, Elena H. Chartoff

**Affiliations:** 1 Dept. of Psychiatry, Harvard Medical School, McLean Hospital, Belmont, Massachusetts, United States of America; 2 Graduate Program in Neuroscience, Harvard Medical School, Boston, Massachusetts, United States of America; 3 Division of Sleep Medicine, Dept. of Medicine, Harvard Medical School, Brigham & Women’s Hospital, Boston, Massachusetts, United States of America; University of Texas Southwestern Medical Center, UNITED STATES OF AMERICA

## Abstract

Opioid dependence is defined by an aversive withdrawal syndrome upon drug cessation that can motivate continued drug-taking, development of opioid use disorder, and precipitate relapse. An understudied but common opioid withdrawal symptom is disrupted sleep, reported as both insomnia and daytime sleepiness. Despite the prevalence and severity of sleep disturbances during opioid withdrawal, there is a gap in our understanding of their interactions. The goal of this study was to establish an in-depth, temporal signature of spontaneous oxycodone withdrawal effects on the diurnal composition of discrete sleep stages and the dynamic spectral properties of the electroencephalogram (EEG) signal in male rats. We continuously recorded EEG and electromyography (EMG) signals for 8 d of spontaneous withdrawal after a 14-d escalating-dose oxycodone regimen (0.5–8.0 mg/kg, 2×d; SC). During withdrawal, there was a profound loss (peaking on days 2–3) and gradual return of diurnal structure in sleep, body temperature, and locomotor activity, as well as decreased sleep and wake bout durations dependent on lights on/off. Withdrawal was associated with significant alterations in the slope of the aperiodic 1/f component of the EEG power spectrum, an established biomarker of arousal level. Early in withdrawal, NREM exhibited an acute flattening and return to baseline of both low (1–4 Hz) and high (15–50 Hz) frequency components of the 1/f spectrum. These findings suggest temporally dependent withdrawal effects on sleep, reflecting the complex way in which the allostatic forces of opioid withdrawal impinge upon sleep and diurnal processes. These foundational data based on continuous tracking of vigilance state, sleep stage composition, and spectral EEG properties provide a detailed construct with which to form and test hypotheses on the mechanisms of opioid-sleep interactions.

## Introduction

Prescription opioid painkillers such as oxycodone exert both analgesic and euphoric effects, the latter of which contribute to high rates of misuse, opioid dependence, opioid use disorder (OUD), and overdose [1]. OUD is a chronic, relapsing disorder characterized by escalating, compulsive drug use despite harmful consequences, a constellation of aversive withdrawal signs, cravings, and relapse [2]. A primary barrier to treatment of OUD is the experience of opioid withdrawal, comprising both acute somatic and protracted affective components [3–5]. Understanding the spectrum of withdrawal signs and their underlying mechanisms is essential to developing effective treatments.

Sleep disturbances are some of the most highly reported, but least studied, withdrawal symptoms for a number of drugs of abuse, including opioids [6–8]. In humans, withdrawal-induced sleep disturbances typically include insomnia, fragmented sleep, and excessive daytime sleepiness [6, 9, 10]. Although the specific influence of withdrawal-related sleep disturbances on patient outcomes remains understudied, they have been associated with increased opioid craving, greater clinical severity, and misuse of benzodiazepines [11–13]. Indeed, the majority of people with OUD entering outpatient treatment report some sleep disturbances [14]. Although several preclinical and clinical studies have assessed the effects of acute opioids on sleep [15–17], there are relatively few studies specifically characterizing the effects of opioid withdrawal on sleep. In the 1970s, Khazan and colleagues performed early studies on REM sleep and EEG voltage output in rats undergoing spontaneous withdrawal from morphine injections [18, 19], which were later expanded in the 2020s in mouse models [20, 21]. These studies showed an overall flattening of the diurnal sleep rhythms typical of rodents, with decreased sleep during the rodents’ typical inactive period (lights-on) and increased sleep during the active period (lights-off), which is broadly consistent with the clinical observation of insomnia and increased daytime sleepiness [20, 21].

Oxycodone activates mu opioid receptors (MOR), which are expressed throughout the brain, including regions that regulate reward, motivation, and arousal [22–24]. Acute MOR activation typically decreases neuronal activity [25], whereas chronic MOR activation results in time-dependent molecular, cellular, and circuit adaptations to counter persistent neuronal inhibition [25–28]. Upon removal of the opioid (i.e., withdrawal), there is an “unmasking” of these previously MOR-suppressed homeostatic changes, resulting in rebound increases in neuronal activity within MOR-expressing cells [25, 26, 28, 29]. Putative interactions between opioid withdrawal and sleep depend, in part, on the neuroanatomical expression of MORs and their homeostatic responses to chronic opioid exposure and withdrawal. Opioid dependence and withdrawal constitute strong internal and external stimuli that modulate diurnal processes [30, 31], homeostatic processes that track sleep need [32–35], and allostatic processes such as stress [36]. As such, there are numerous possibilities through which opioid withdrawal can influence sleep-wake regulatory networks.

Systems-level neural activity during sleep is commonly measured through the electroencephalogram (EEG), which represents the aggregate dynamics of numerous cortical and subcortical networks over time [37, 38]. Sleep architecture in rodents is categorized into wake, rapid eye movement (REM) sleep, and non-REM (NREM) sleep based on a standardized combination of behavior and EEG events [37]. Thus, sleep EEG analysis can serve as a means of characterizing the effects of opioid withdrawal on sleep dynamics, as well as specific effects on the neural mechanisms governing sleep processes [37, 39, 40]. Broadly, the EEG signal can be decomposed into periodic (oscillatory) and aperiodic (non-oscillatory) components. Periodic components are derived from synchronized activity within and between different brain networks and generally manifest as salient spectral peaks within canonical frequency bands: delta (<4 Hz) related to NREM sleep and homeostatic drive, theta (4–8 Hz) related to hippocampal processing and REM power in rodents, alpha (8–12 Hz) related to arousal and wakefulness, and beta (12–30 Hz) and gamma (>30 Hz) related to cognitive processing, learning, and memory [37]. In contrast, it is thought that aperiodic components of the power spectrum density (PSD) do not originate from any regular, rhythmic process, but rather from the integration of synaptic currents [41].

While the structure of the aperiodic component is complex [42, 43], it is typically described in terms of a simplified single, or piecewise, power-law model (1/*f*^*α*^), in which spectral power decays as the frequency, *f*, increases, modulated by a time constant of decay, *α* [44]. The decay constant is most commonly estimated as the slope of the linearized log-log transformed EEG power spectrum. Piecewise models will fit this constant separately in high and low frequency components, which are separated by a pivot point (at ~20–30 Hz in humans) often termed a “knee” [43, 45]. Although the underlying neural mechanisms responsible for the aperiodic component of the EEG are not fully understood, changes in aperiodic slope have been correlated with excitatory-inhibitory (EI) synaptic balance [43, 46]. Increasing evidence shows that pharmacological excitation of neural activity is associated with flatter aperiodic slopes, whereas inhibition is associated with steeper slopes [45, 47]. As such, tracking the aperiodic slope throughout oxycodone withdrawal may provide a temporal signature of changes in global neural activity and arousability.

In this study, we implanted rats with wireless telemetry devices that continuously capture EEG, EMG, body temperature, and locomotor activity without restricting movement or posture, enabling a comprehensive and temporal analysis of how spontaneous oxycodone withdrawal alters sleep states and their associated EEG spectral dynamics in freely moving animals. These foundational studies aim to establish signatures of opioid withdrawal on sleep processes that will engender future mechanistic studies and may ultimately be used to inform the development of novel therapeutics targeting sleep disruptions.

## Materials and methods

### Animals

Adult male Sprague Dawley rats (Charles River Laboratories, Wilmington, MA]) weighing 250-275g upon arrival were initially group-housed in the animal care facility. After surgery, rats were individually housed in a satellite “sleep room” designated specifically for rodent sleep studies, with each cohort of rats being the sole occupants of the sleep room. Both the animal care vivarium and sleep room were kept on a 12h light/dark (7:00 AM lights on / 7:00 PM lights off) cycles. Standard rat chow (Lab Diet 5012) and water were available *ad libitum*. A total of 16 rats were tested: 12 rats received 14-d of oxycodone infusions and 4 rats received 14-d of saline infusions. To ensure the reproducibility of our data, 16 rats in this study were divided among 5 independent cohorts, tested independently and sequentially. The 16th rat (saline) was taken from a parallel study using similar methods (see below; Extra Saline Rat). Cohort 1 (N = 4 oxycodone), Cohort 2 (N = 3 oxycodone), Cohort 3 (N = 1 oxycodone), Cohort 4 (N = 3 oxycodone), Cohort 5 (N = 1 oxycodone; 3 saline), and the Extra rat (N = 1 saline). 15 out of 16 rats had their sleep data analyzed; one oxycodone rat from cohort 1 (rat A5) had poor signal quality, so its sleep data was not scored or analyzed, however, its somatic withdrawal behavior was scored.

### Extra saline rat

Note that in Cohort 5, 1 of the 3 saline rat recordings showed non-physiological, harmonic noise at higher frequencies which corrupted the EEG power spectrum. Therefore, we did not include data from this saline rat in our final EEG dynamics analyses, but instead added a chronic saline-treated rat from a separate experiment testing the effects of suvorexant on sleep. This additional saline rat received 14 days of saline exactly as described for this study, followed by a SC injection of 100% DMSO (vehicle for suvorexant) on equivalent days of W2, W3, and W4 at around ZT0. The housing and treatment of rats were given written approval by the McLean Hospital Institutional Animal Care and Use Committee and followed guidelines set by the National Institutes of Health.

### Surgeries

At least one week after arrival, rats underwent surgery (body weight at time of surgery was 310-330g). Briefly, rats were anesthetized with isoflurane (Covetrus; isoflurane at 4–5% for induction, 1.5–3% for anesthesia maintenance; 1.5L/min O_2_ flow) and subcutaneously implanted with both a telemetry device [HD-S02; Data Sciences International (DSI)] and a programmable infusion pump [iPrecio SMP-200; DURECT Corporation (iPrecio pump)].

#### Telemetry surgeries

Anesthetized rats were implanted with the wireless contained telemetry device, with 4 biopotential leads: 2 EMG leads that comprise one EMG channel and 2 EEG leads that comprise one channel. For each implant, the EMG leads were threaded through the cervical trapezius muscle via a small incision made with a 20-gauge needle and was anchored to the muscle using non-dissolvable silk sutures. The EEG leads were individually secured to two skull screws (Plastics One, cat #00–80 x 1/16; 1.6mm long) that contacted dura. The negative EEG lead was secured to the screw at +2.0mm anteroposterior and at lateral left 1.5mm, and the positive EEG lead was secured to the screw at -7.0mm anteroposterior and lateral right -1.5mm–all relative to Bregma. The screw plus lead assemblies were held in place on the skull with dental cement (Ortho-Jet Package; Lang Dental).

#### iPrecio drug pump surgeries

Immediately after implanting the telemetry device, iPrecio drug pumps were subcutaneously implanted. The pump tubing, from which drug is released, was cut to 2.5–3.0cm prior to implantation. A subcutaneous pocket was created at 3-4cm posterior to the scapula and 2cm lateral and parallel to the spine, and the pre-programmed iPrecio pump was inserted. To secure the pump within the pocket, a suture was threaded through the underlying external oblique muscle and then through a suture anchor located on the pump. After surgery, rats were treated one time with the non-steroidal anti-inflammatory drug (NSAID) Ketoprofen (5 mg/kg, IP) and individually housed in the sleep room.

### Physiological telemetry recordings

**Fig 1:**
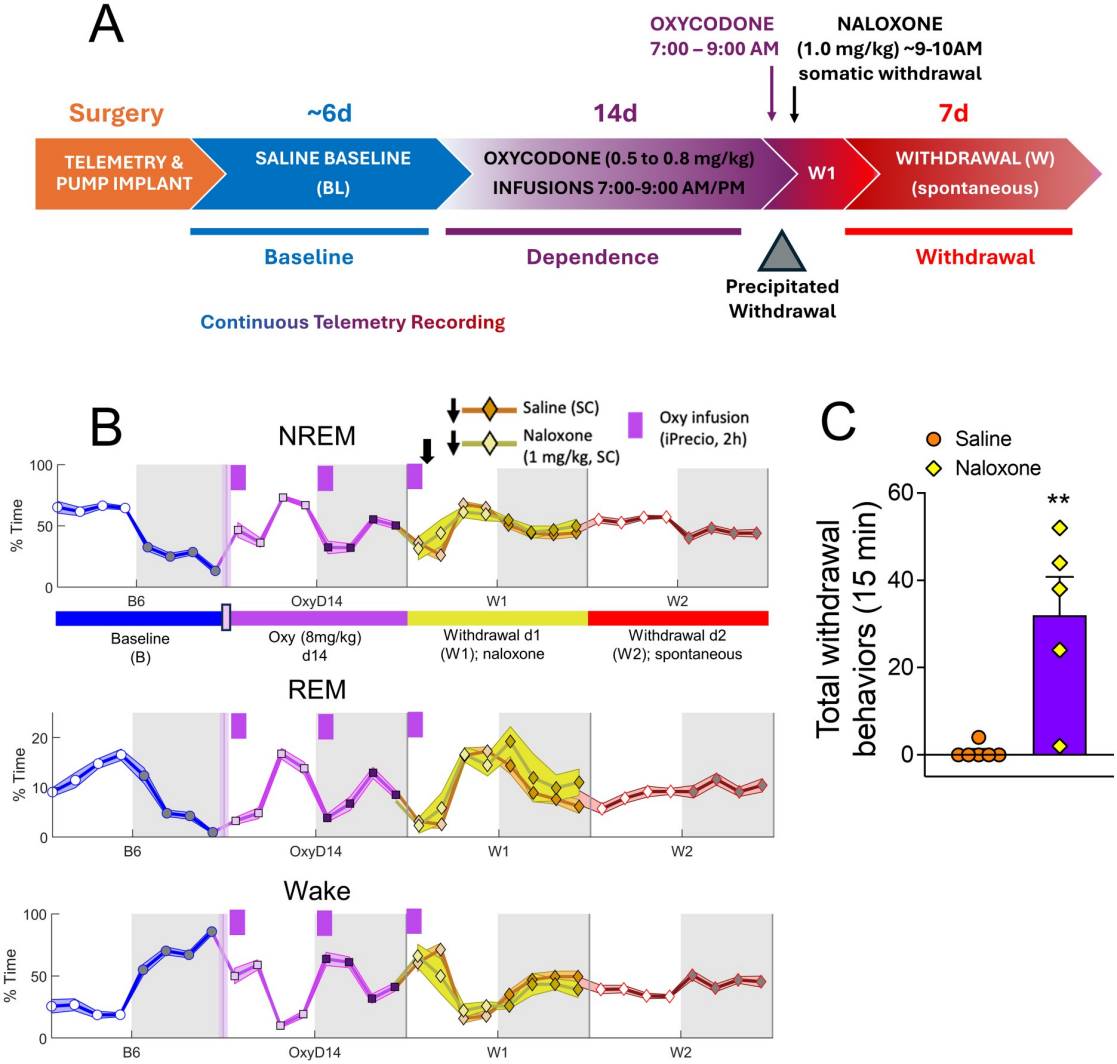
Escalating oxycodone dose infusion protocol modulates sleep stages and produces dependence in male rats. (**A**) Experimental schematic. Rats were implanted subcutaneously (SC) with both a telemetry device and a programmable infusion pump. After recovery, pumps infused saline for 5 days, the last 3 of which were used as baseline sleep recordings. Following saline infusions, pumps delivered escalating-dose oxycodone twice a day (ZT0-2 and ZT12-14) for 14 days (0.5–8.0 mg/kg/inf, 2 inf/d), resulting in 28 total infusions. Starting with 0.5 mg/kg, rats received the following: number of infusions at oxycodone dose: 4 at 0.5, 6 at 1.0, 6 at 2.0, 6 at 4.0, 6 at 8.0. The last infusion (8.0 mg/kg) occurred from ZT0-2 on withdrawal day 1 (W1). Immediately after the last infusion, rats were injected with either saline or naloxone (1.0 mg/kg, SC). (**B**) % time in each sleep stage (NREM, REM, Wake) presented as average % time ± SEM in 3-h bins for baseline day 6 (B6), oxycodone day 14 (8 mg/kg; OxyD14), withdrawal day 1 (W1) divided into saline- (orange diamonds; N = 7) or naloxone- (yellow diamonds; N = 4) treated data, and withdrawal day 2 (W2). (**C**) Somatic withdrawal scores from oxycodone-dependent rats on W1, quantified from video recordings 20–35 minutes post-naloxone (yellow diamonds) or saline (orange circles) injections. Total withdrawal behaviors are the sum of ptosis, flattened posture, rats putting their jaw/snout to the surface or bedding of the cage and stereotyped head bobs instances in the 15-min period. **p<0.01, unpaired t-test, comparing oxycodone-dependent rats injected with saline (N = 6) or naloxone (N = 5). Underlying data is in “Fig1_Data in S1 File”.

Three to five days after surgery, telemetry devices were activated by a magnet (DSI), which puts the devices on “standby”, allowing control via DSI Ponemah software. Receivers (RPC-1 PhysioTel; DSI) that detect radio signal are placed underneath each rat cage to detect AM signals emitted by the telemetry transmitters. Data was recorded 24 h/d throughout the experiment (see Fig 1A) and included 500-Hz interpolated sampling of EEG and EMG and 10-s bins of mean subcutaneous temperature and activity counts. Zeitgeber time 0 (ZT0) was defined as 7:00AM. EEG, EMG, motor activity, and temperature were analyzed using NeuroScore (Data Science International, St. Paul, MN).

### Escalating-dose oxycodone regimen to induce dependence

#### Oxycodone administration

Prior to implantation, iPrecio pumps were programmed using the iPrecio Management System and Software (IMS-200; ALZET) to infuse either saline or oxycodone twice a day for two hours each (ZT0-2 and ZT12-14). For the first several days after surgery, the pumps infused 0.9% saline for 2h. Fig 1A shows a schematic of the experimental design. Telemetry data from the last 3 days of saline infusions were used as baseline. Following baseline, saline was removed from the pumps and replaced with oxycodone (0.5 mg/kg). The timeline for escalating oxycodone doses included a total of 28 oxycodone infusions (ZT0-2 and ZT12-14 each day). On oxycodone day 1 (Oxy d1), rats received saline in the morning (ZT0-2) and oxycodone (0.5 mg/kg) in the evening (ZT12-14). On the last drug day, Oxy d15, rats received oxycodone (8.0 mg/kg) in the morning (ZT0-2) and no infusion in the evening (ZT12-14). Between Oxy d1 and Oxy d15, rats received 24 additional infusions of oxy (0.5 mg/kg), 6 infusions of oxy (1.0 mg/kg), 6 infusions of oxy (2.0 mg/kg), 6 infusions of oxy (4.0 mg/kg), and 5 infusions of oxy (8.0 mg/kg), with the 6^th^ oxy (8.0 mg/kg) infusion occurring on Oxy d15 (ZT0-2). For all cohorts except cohort 2, infusions were delivered at rates of 15μl/h (30 μl infused) for the first 4 infusions and 30μl/h (60 μl infused) for all remaining infusions. Cohort 2 received saline during baseline and the oxy 0.5mg/kg infusions at 7.4μl/hr, oxy 1.0mg/kg at 14.8μl/hr and all remaining infusions at 29.8μl/hr. Separate rats received saline throughout the 14-d pump delivery regimen and served as controls for any effects of surgeries, interoceptive effects of infusions, isoflurane exposures, and aging/weight gain on sleep. In addition, saline rats controlled for telemetry implant and receiver stability over the course of the experiment.

The iPrecio pumps were emptied and refilled with the appropriate concentration of oxycodone (or saline) 4 times, always between ZT7-12. Oxycodone concentrations were calculated based on individual rat weights, the volume infused, and the rate of infusion. To refill the pumps, rats were briefly anesthetized with 4–5% isoflurane, and a 26-gauge syringe was used to withdraw all fluid in the reservoir and add new drug via a port on the pump that could be felt through the skin. The volume of drug withdrawn during a pump change served as a control that the pumps were delivering the expected volume of drug.

#### Oxycodone withdrawal

To demonstrate that the escalating-dose oxycodone regimen produced dependence, rats were injected with the opioid receptor antagonist, naloxone (1.0 mg/kg, SC), or saline, within an hour (9–10 AM) (after the last oxycodone (8.0 mg/kg) infusion ended on Oxy d15 [referred hereafter as withdrawal day 1 (W1)] to assess naloxone-precipitated somatic withdrawal signs (with the exception of rat A36 who received a saline injection at 11 AM). Immediately after naloxone (or saline) injections, rats were videotaped in their home cages for at least 35 minutes, and minutes 20–35 of the videos were subsequently scored for withdrawal signs by a trained experimenter, as previously described [27]. Briefly, videos were scored for somatic withdrawal signs including ptosis, flattened posture, rats putting their jaw/snout to the surface or bedding of the cage, and stereotyped head bobs (where the rat is sitting down and sniffs the air or moves only their head). Scoring was standardized such that every 15 seconds, the scorer would glance at the video and note which behavior a rat was doing at that moment. To derive a Total Withdrawal score, the instances of each recorded behavior were summed over the 15 minutes. Because rats received either naloxone or saline injection on W1, we did not consider sleep data collected on that day to cleanly represent spontaneous withdrawal. Rather, ZT0 on W2 marked the start of spontaneous oxycodone withdrawal in our study. 11 out of the 12 oxycodone rats had their W1 behavior analyzed (see Fig1_Data in S1 File).

#### Sleep scoring

Sleep was manually scored by a trained experimenter in 10-second epochs by visually inspecting the EEG and EMG signal combined with a spectral analysis of the EEG waveform using NeuroScore software. Using a standard, objective set of rules based on a combination of behavior and EEG events, sleep states were categorized into wake, rapid eye movement (REM) sleep, and non-REM (NREM) sleep (Missig et al., 2018). NREM sleep was characterized by the presence of high amplitude and low frequency waves in the EEG combined with relatively little activity in the EMG. Wake was characterized by low amplitude EEG combined with relatively high activity in the EMG and locomotor activity. REM sleep was characterized by the dominance of theta waves (4-8Hz). Sleep stage scoring was done by the same experimenter who handled the 14-d drug infusion phase of the experiment.

### Diurnal metrics

The daily rhythms of sleep, temperature, and activity result from interactions between homeostatic and diurnal processes [33]. In this study, we refer to all sleep and physiological rhythms analyzed as “diurnal rhythms”, meaning they occur within a 24-h day/night period. Percent time in Wake, NREM, and REM as well as activity counts and temperature were computed for 3 baseline (BL) days and for withdrawal days 2–8 (W2-W8). During the first several days of oxycodone withdrawal, there was a clear, qualitative reduction in the nocturnal amplitudes of our behavioral measures: Wake, NREM, REM, activity, and temperature. Therefore, we computed a diurnal index (DI) for BL and W2-W8 for each measure. We defined the DI for a given measure as the difference between the maximum 3h bin value and the minimum 3h bin value on a particular day relative to each individual rat’s average baseline difference.

### Spectral estimation

To characterize changes in neural activity during withdrawal, we performed spectral estimation on the EEG data, which provides a basis for quantifying the frequency content of the EEG signal. Spectral estimation was performed using the multitaper approach, which is an estimator with optimized bias and variance properties [48] commonly used for sleep EEG analyses [39]. Multitaper spectrograms were computed with the following parameters: frequency range: 0.5–50Hz, time-bandwidth product: 5, number of tapers: 9, window size: 10s, window step: 5s. For analyses of specific conditions or time segments, fixed spectra were computed by averaging the spectrogram over the given period. See Prerau et. al (2017) [39] for a detailed discussion of multitaper parameter selection. To adjust for inter-rat variability in recording, we computed relative power, defined as the power within each frequency bin divided by the total power between 0.5 and 50Hz, which was log-transformed for analysis.

### Parameterizing the aperiodic slope

While numerous sophisticated approaches have been proposed to model the non-linear aspects of the aperiodic EEG spectrum [44, 49], the most straightforward method transforms the spectrum into the log-log space, in which the decay constant can be parameterized as the slope of a line. This linear approach has been shown to have a negligible deviation in population estimates relative to the non-linear approaches [44]. We fit the piecewise low (1-4Hz) and high (15-50Hz) components of the log-log transformed spectra using least-squares linear regression with an offset, from which the value of the slope parameters was used as the estimate. It should be noted that there is no scientific consensus for the frequency range of the high and low components, which are determined purely ad-hoc based on the application and whether human or animal models are being used [45, 46, 47].

It should be noted that 3 out of 11 oxycodone rat recordings showed non-physiological, harmonic noise at higher frequencies which corrupted the EEG power spectrum, biasing the aperiodic spectral estimation. This noise can be caused by a myriad of factors, including the integrity of the recording lead, as well as external machine noise. Rather than relying on noise removal algorithms which may produce biased interpolation of the missing data, we conservatively chose to exclude data from these three oxycodone rats in our final EEG dynamics analyses.

### Experimental design and statistical analyses

Statistical comparisons were performed using GraphPad Prism version 10 (GraphPad Software). For all telemetry data, we used a within-subjects design in which each rat’s withdrawal data was compared to the average of data from baseline days 4, 5, and 6 (see Schematic, Fig 1). We considered spontaneous withdrawal to start at ZT0 on W2, because this was the first full withdrawal day not confounded by additional drug treatments (i.e. on W1, rats received their last oxycodone infusions from ZT0-2 followed by either an acute injection of naloxone or saline at ZT0.5).

In general, the normality assumption does not hold for % Time, temperature, activity, DI, bout #, bout length, relative power, and 1/f slopes (verified by D’Aostino and Pearson, Anderson-Darling tests). We therefore conservatively used the nonparametric Friedman’s Test on these data, followed by Dunn’s multiple comparison tests for analysis of these data, which are compared to BL_avg_. For simplicity, we used 2-way, repeated measures ANOVA followed by Dunnett’s multiple comparison tests for the 3-h time bin comparisons. Significance level was set at *p*<0.05.

The first cohort of rats (N = 3) missed the first two oxycodone 0.5mg/kg infusions and received two extra infusions of saline instead. However, they received the rest of the scheduled 26 escalating oxycodone infusions: 2 infusions of oxy 0.5mg/kg, 6 infusions of 1mg/kg, 6 infusions of 2mg/kg, 6 infusions of 4mg/kg, and 6 infusions of 8mg/kg. As such, we included this cohort within our analysis.

**Fig 2:**
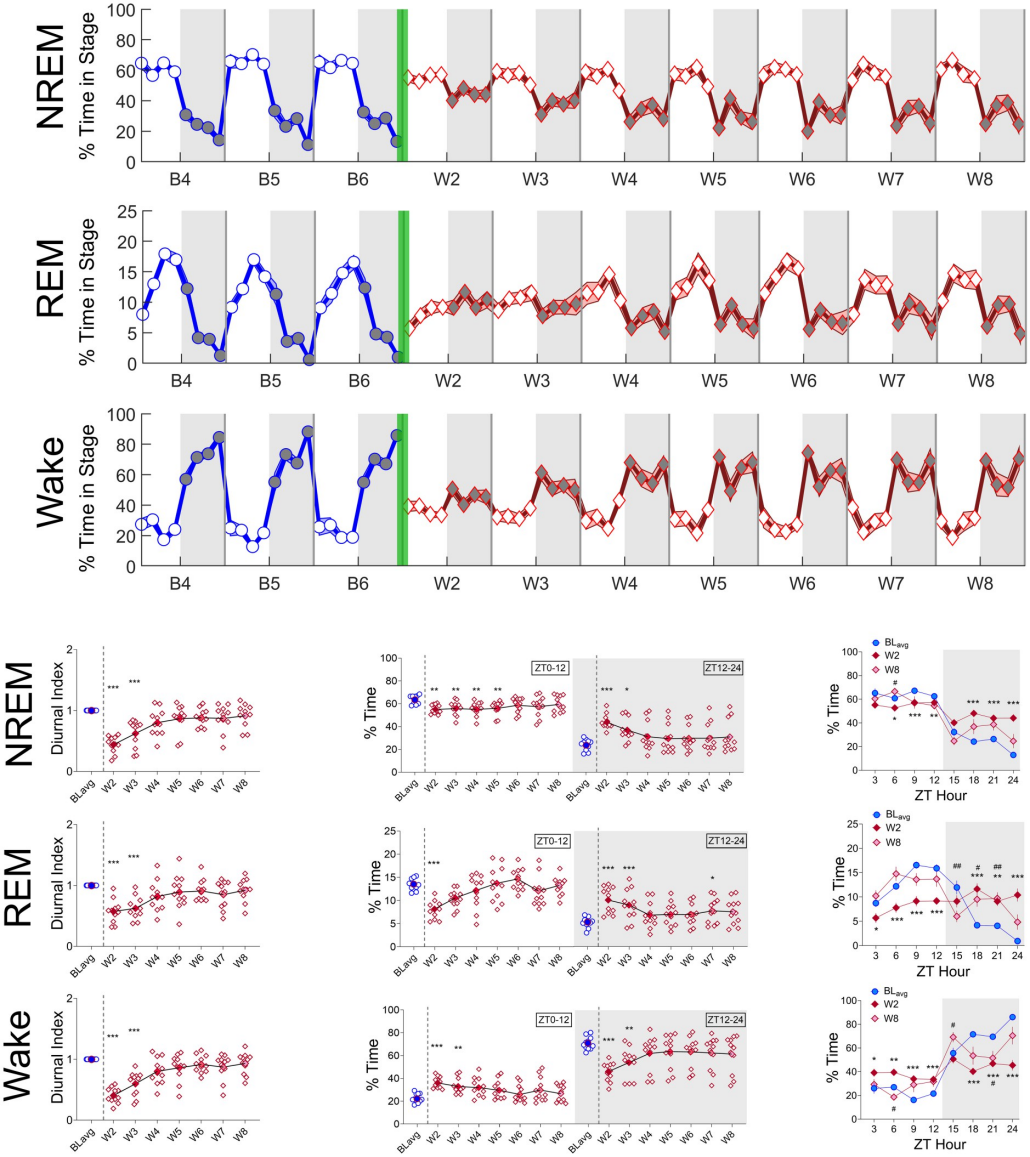
Spontaneous oxycodone withdrawal transiently flattens diurnal sleep rhythms. The mean % of total time in 3-h bins (±SEM) spent in each sleep stage [(**A**) NREM, (**B**) REM, and (**C**)Wake] is shown for baseline days 4, 5, and 6 (B4, B5, B6; blue line, circles) and spontaneous withdrawal days 2–8 (W2-W8; red line, diamonds). Vertical green bars between B6 and W2 represent the 14-d escalating oxycodone dose regimen and W1. Grey shaded regions represent ZT12-24 (lights off), and unshaded regions represent ZT0-12 (lights on). For NREM, REM, and Wake (**D, E, F**, respectively), the mean (±SEM, with individual rat data) Diurnal Index (DI; left panels), % total time (ZT0-12 or ZT12-24; center panels), and the mean (±SEM) % of total time in 3-h bins for BL_avg_, W2, and W8 (right panels) are shown. DI is defined as each day’s maximum—minimum 3-h bin difference divided by the individual rat’s average baseline difference. For D, E, F, left and middle panels: *p<0.05, **p<0.01, ***p<0.001, Dunn’s post hoc tests. For D, E, F, right panels, *p<0.05, **p<0.01, ***p<0.001 comparing W2 to BL_avg_, and #p<0.05, ##p<0.01 comparing W8 to BL_avg_, Dunnett’s post hoc tests. N = 11 rats. Underlying data is in Fig2_Data in S1 File. Abbreviations: ZT, zeitgeber time; BL_avg_, average of baseline days 4, 5, 6; W, withdrawal.

**Fig 3:**
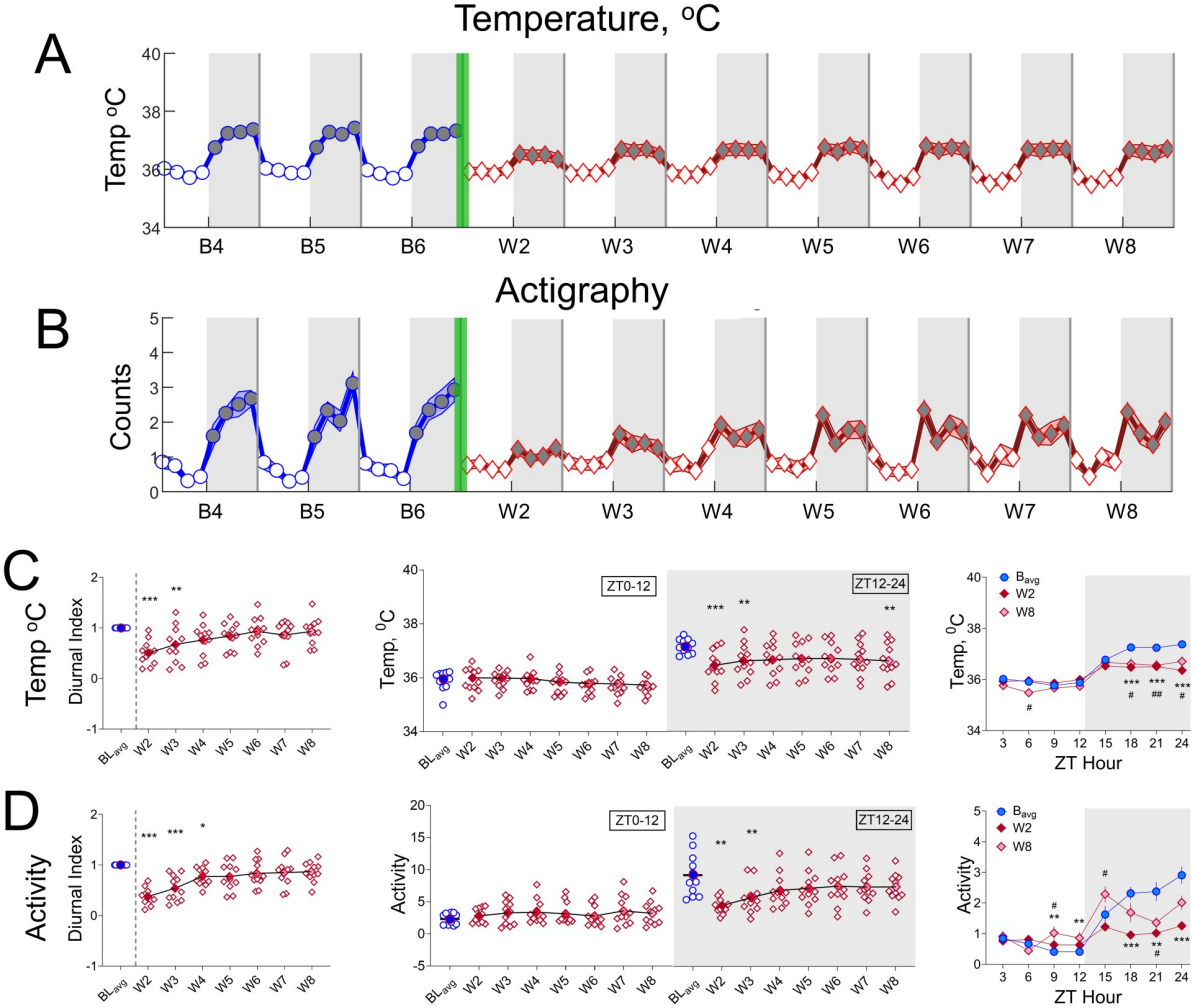
Spontaneous oxycodone withdrawal transiently reduces temperature and activity during lights off. The mean temperature (°C; A) and activity counts (in thousands) (B) in 3-h bins (±SEM) are shown for baseline days 4, 5, and 6 (B4, B5, B6; blue line, circles) and for spontaneous withdrawal days 2–8 (W2-W8; red line, diamonds). Vertical green bars between B6 and W2 represent the 14-d escalating oxycodone dose regimen and W1. Grey shaded regions represent ZT12-24 (lights off), and unshaded regions represent ZT0-12 (lights on). For temperature and activity (C, D, respectively), mean (±SEM, with individual rat data) Diurnal Index (DI; left panels), % total temperature (C, middle panel) or activity counts (in hundreds) (D, middle panel) during ZT0-12 or ZT12-24, and mean (±SEM) temperature (C, right panel) or activity counts (D, right panel) in 3-h bins for BL_avg_, W2, and W8 are shown. For C, D, left and middle panels: *p<0.05, **p<0.01, ***p<0.001, Dunn’s post hoc tests. For C, D, right panels, **p<0.01, ***p<0.001 comparing W2 to BL_avg_, and #p<0.05, ##p<0.01 comparing W8 to BL_avg_. N = 11 rats. Underlying data is in Fig3_Data in S1 File. *Abbreviations*: ZT, zeitgeber time; BL_avg_, Baseline average; W, withdrawal.

**Fig 4:**
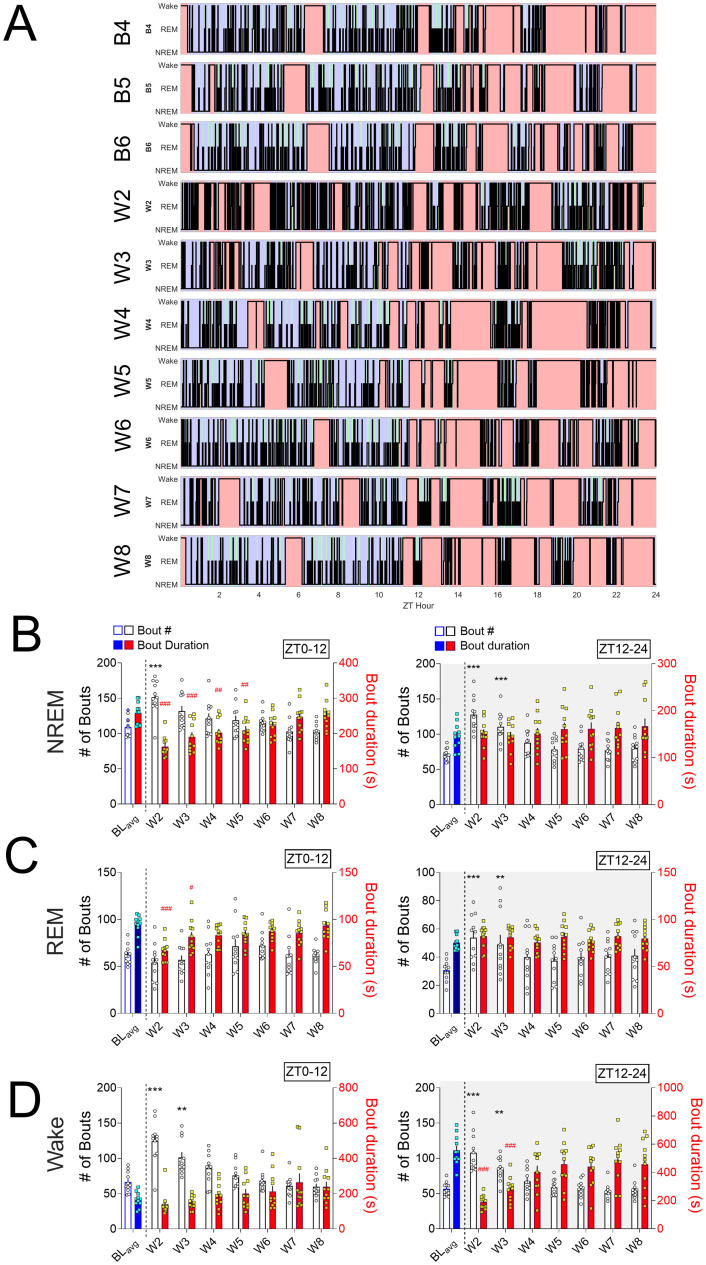
Spontaneous oxycodone withdrawal disrupts sleep/wake architecture. (**A**) Hypnogram data from a representative oxycodone-treated rat is plotted for each of B4-B6 and W2-W8 days, with Wake represented at the top of each hypnogram with red shading, REM in the middle with green shading and NREM at the bottom with blue shading. For NREM, REM, and Wake (**B, C, D**, respectively) the mean (±SEM, with individual rat data) number of bouts (white bars, left Y axis) and bout durations (filled bars, right Y axis) are shown for BL_avg_ and W2-W8 for ZT0-12 (**left panels**) and ZT12-24 (**right panels**). For lights on, when rats show less % time asleep, the number of NREM (**B, left panel**) and Wake (**D, left panel**) bouts is increased, with corresponding decreases in NREM and REM bout duration. For lights off, there is a significant increase in the # of bouts of all sleep stages (**B, C, D, right panels**) with a corresponding decrease in Wake bout duration (**D, right panel**). *p<0.05, **p<0.01, ***p<0.001, comparing bout # during withdrawal to BL_avg_ bout #, Dunnett’s post hoc tests. #p<0.05, ##p<0.01, ###p<0.001 comparing bout duration during withdrawal to BL_avg_ bout duration, Dunnett’s post hoc tests. N = 11 rats. Underlying data is in Fig4_Data in S1 File. *Abbreviations*: ZT, zeitgeber time; Baseline (B4, B5, B6 average), BL_avg_; W, withdrawal.

From observation of the data, one cohort (cohort 4) showed prolonged withdrawal effects which made them outliers in later withdrawal days. To further explore if and how the data from Cohort 4 rats impacted our overall results, we analyzed a subset of data from all but the 3 rats from Cohort 4 and compared these results to those shown in: DI data from % time in NREM, REM, and Wake, temperature, and activity (Figs 2D–2F, 3C and 3D) and, NREM bout numbers and durations data (Fig 4B), and NREM relative power for Delta and Gamma frequency bands (Fig 6A, 6E). In each case, there was no effect of Cohort 4 rats on the statistical significance of the results. For DI data from % time in NREM, REM, and Wake, temperature, and activity, Friedman’s nonparametric tests followed by Dunn’s multiple comparisons tests were used to compare withdrawal days to BL_avg_ for the N = 8 group (no Cohort 4). NREM: Q = 26.16, p = 0.0005; REM: Q = 33.40, p<0.0001; Wake: Q = 32.39, p<0.0001; Temp: Q = 25.42, p = 0.0006; Activity: Q = 33.58. Multiple comparison’s tests showed that the DI for NREM, REM, Wake, and Activity was significantly decreased from the respective BL_avg_ on W2 and W3, which is identical to results obtained when all 11 rats were analyzed (Figs 2D–2F and 3D). The only difference was in the DI for Temperature, in which only W2, rather than W2 and W3, was significantly different compared to BL_avg_ in the N = 8 group. For NREM relative power for Delta and Gamma frequency bands, two-way repeated measures ANOVAs followed by Dunnetts’s multiple comparisons tests were used to compare 3-h bin data from W2 and W8 to BL_avg_ for the N = 8 group (no Cohort 4). Similar to analyses for all 11 rats, there were significant Time bin X Withdrawal Day interactions for Delta: F_(4.135, 28.95)_ = 14.93, p<0.0001 and Gamma: F_(3.374, 23.61)_ = 20.83, p<0.0001. Multiple comparison’s tests for the N = 8 data (no Cohort 4) showed identical pairwise significance as that shown in Fig 6A, 6E (left panels). As such, data from cohort 4 were included in the analyses for this report, as there was no justification to exclude them.

## Results

### Experimental overview

To track the effects of spontaneous withdrawal from chronic oxycodone administration on sleep/wake, rats were subcutaneously implanted with telemetry devices to capture EEG and EMG, temperature, and activity signals and subcutaneous drug pumps to deliver saline or oxycodone (Fig 1A). Pumps were programmed to deliver drug twice a day (ZT0-2, lights-on and ZT12-14, lights-off). Telemetry data was continuously collected throughout the experiment. The telemetry data from the last 3 days of saline administration (Fig 1A, blue chevron) were used as baseline. Escalating-dose oxycodone was delivered for 14 days, with the last infusion occurring from ZT0-2 on withdrawal day 1 (W1). This enabled testing for naloxone-precipitated withdrawal 30–45 minutes after the last oxycodone infusion ended at ZT2.

Rats have a nocturnal sleep/wake rhythm in which most of their sleep (NREM and REM) occurs during the day (lights-on = ZT0-12; subjective day), and most of their Wake occurs during the night (lights-off = ZT12-24; subjective night) [50, 51]. This typical nocturnal sleep/wake rhythm is observed at baseline (B; Fig 1B, blue), whereas the ZT0-2 oxycodone infusion on Oxy d14 suppresses sleep (NREM, REM) and increases Wake for the first 6 hours of lights on (ZT0-6) (Fig 1B, purple). Oxycodone infusions from ZT12-14 (lights off) do not themselves alter the percent time in sleep stage relative to baseline, but rather the offset of oxycodone effects (ZT18-24) is associated with increased sleep and decreased wake (Fig 1B, purple). It is important to highlight that telemetry data from each oxycodone infusion over the course of the 14-d escalating dose regimen have been recorded but are not the focus of this paper. Here we are specifically interested in how oxycodone withdrawal impacts sleep.

This study focuses primarily on the temporal effects of spontaneous oxycodone withdrawal measured over withdrawal days 2–8 (W2-W8), (Fig 1A, red chevron), using naloxone-precipitated withdrawal for comparison. Averaged % time in 3-h bins over 24-h periods in each sleep stage (NREM, REM, Wake; Fig 1B) is shown for each of the 4 stages of the experiment: baseline (B, blue), oxycodone (purple), W1 (naloxone, yellow; naloxone, orange), and spontaneous withdrawal W2-W8 (red). Qualitatively, the % time in each sleep stage on W1 appears similar for rats treated with naloxone or saline, suggesting minimal differences between the effects of precipitated withdrawal and spontaneous withdrawal on sleep. Oxycodone dependence on W1 was confirmed by measuring naloxone-precipitated somatic withdrawal behaviors (Fig 1C).

#### Spontaneous oxycodone withdrawal transiently flattens sleep-wake rhythms

To characterize the effects of spontaneous oxycodone withdrawal on the diurnal rhythmicity of sleep architecture observed during baseline recording days, we plotted the percent of total time spent in NREM, REM, and Wake (Fig 2A–2C, respectively) in 3-h bins covering the last 3 days of baseline recordings (saline infusions, B4, B5, B6) and spontaneous oxycodone withdrawal days 2–8 (W2-W8). Withdrawal day 1 (W1) is not included in our spontaneous withdrawal results because rats received their last 2-h infusion of oxycodone and an injection of either naloxone or saline on the morning of W1 (see Methods). For all sleep states, we observed typical diurnal rhythmicity during B4-B6, followed by a marked flattening (loss of rhythmicity) during early withdrawal (~W2-W4; Fig 2A–2C) and a return towards baseline patterns by W8. To quantify the effects of spontaneous oxycodone withdrawal on diurnal rhythmicity during NREM, REM, and Wake, we compared the magnitude and timing of several sleep stage features during W2-8 to the average of the last 3 baseline days (BL_avg_). First, we computed the diurnal index (DI), defined here as the peak-to-trough amplitude in the 3-h bin values on a particular day, normalized to each individual rat’s average peak-to-trough amplitude over the last 3 baseline days (BL_avg_). A decrease in DI relative to BL_avg_ indicates a reduction in the magnitude of diurnal rhythmicity (i.e., flattening). For the % of time in each sleep state, Friedman’s Tests show that the DI is significantly reduced compared to BL_avg_: [NREM, Q = 44.95, p<0.0001, Fig 2D, left; REM, Q = 31.44, p<0.0001, Fig 2E, left; Wake, Q = 45.18, p<0.0001, Fig 2F, left). To control for the possibility that rats injected with naloxone on W1 (1.0 mg/kg, SC; N = 4) would show different spontaneous oxycodone withdrawal sleep effects compared to rats injected with saline on W1 (1.0 ml/kg, SC; N = 7), we conducted 2-way ANOVAs with repeated measures on withdrawal day for W2-W8 on naloxone vs saline rats. There were no Treatment x Day interactions or Treatment main effects for any sleep stage (S1A), indicating no effect of W1 naloxone injection on subsequent spontaneous oxycodone withdrawal effects on sleep architecture.

To determine whether the effects of oxycodone withdrawal on sleep/wake occur primarily during lights on or off, we quantified the average percent time spent in NREM, REM and Wake for ZT0-12 and for ZT12-24 (Fig 2D–2F, middle panels) and compared W2-W8 to BL_avg_. Overall, we find that during early withdrawal the % time asleep decreases, whereas the % time awake increases, during lights on (NREM, Q = 22.24, p = 0.0023, Fig 2D, middle; REM, Q = 40.55, p<0.0001, Fig 2E, middle; Wake, Q = 28.83, p = 0.0002, Fig 2F, middle). In contrast, there is a general increase in % time asleep and a decrease in % time awake during lights off (NREM, Q = 33.25, p<0.0001, Fig 2D, middle gray; REM, Q = 30.18, p<0.0001, Fig 2E, middle gray; Wake, Q = 33.36, p<0.0001, Fig 2F, middle gray). Together with the DI data, these findings indicate that the most robust effects of spontaneous withdrawal from 14 days of chronic, escalating-dose oxycodone occur during W2 and W3. Further, there is an association between loss of diurnal rhythmicity and insomnia-like reductions in time asleep and subsequent increases in lights off (active period) time asleep.

Since our data suggest a robust flattening of diurnal rhythmicity for each sleep stage on W2 with day-by-day incremental improvements toward baseline, we performed a more granular analysis on the % time in each sleep stage on W2 and W8 by examining the data in 3-h bins and comparing the results to BL_avg_ (Fig 2D–2F; right panels). As expected, the % time in each sleep stage on W2 is relatively flat from ZT0-ZT24, with little difference between lights on and off. On W8, however, recovery towards the rhythmicity of BL_avg_ is observed during lights on, but not lights off. Indeed, similar to W2, the % time in REM and Wake on W8 during several 3-h bins in lights off is significantly different from BL_avg_. These results depend on significant interactions (Treatment day × ZT hour) from 2-way repeated measures ANOVA tests followed by Dunnett’s multiple comparison tests: REM, F_(4.995, 49.95)_ = 13.21, p<0.0001, Fig 2E, right panel; Wake, F_(3.339, 33.39)_ = 11.50, p<0.0001, Fig 2F, right panel.

Like effects on sleep architecture rhythmicity, spontaneous oxycodone withdrawal reduces the magnitude of activity and temperature diurnal rhythmicity (Fig 3A and 3B, respectively), with the greatest effects occurring on W2 and W3 and subsequent recovery towards BL_avg_ by W8. For temperature and activity, Friedman’s Tests show that the DI is significantly reduced compared to BL_avg_: Temp, Q = 34.85, p<0.0001, Fig 3C, left panel; Activity, Q = 48.52, p<0.0001, Fig 3D, left panel. In contrast to the effects of oxycodone withdrawal on sleep; effects on activity and temperature rhythmicity occurred primarily during lights off. This is particularly evident in Fig 3C and 3D, right panels, in which 2-way repeated measures ANOVAs show significant Treatment day × ZT hour interactions (Temp, F_(3.293, 32.93)_ = 7.381, p<0.001, Fig 3C, right panel; Activity, F_(2.742, 27.42)_ = 7.953, p<0.001, Fig 3D, right panel), and Dunnett’s post hoc tests show that significant pairwise comparisons occur during lights off.

#### Both sleep and wake show decreased bout durations during early spontaneous oxycodone withdrawal

Above, we demonstrate that during early spontaneous withdrawal there are overall reductions in the amplitudes of diurnal rhythms for each sleep stage, temperature, and activity, which result in overall changes in the amount of sleep/wake during lights on and off. We next investigated whether oxycodone withdrawal affects sleep stage switching, either through increased or decreased average sleep/wake bout durations. Hypnograms (experimenter-scored sleep stages plotted over time; Fig 4A) are shown for B4-B6 and W2-W8 from a representative oxycodone-treated rat. The hypnograms show marked differences between baseline days and W2 and 3. Qualitatively, the long-duration wake bouts predominant during lights-off decrease in length on W2, replaced with an increased number of sleep bouts, suggesting disrupted sleep/wake architecture on W2 and W3 relative to baseline.

To characterize the degree to which spontaneous oxycodone withdrawal alters sleep/wake architecture, we quantified bout number and bout length (in seconds) for each sleep stage during either lights-on (Fig 4B–4D; left panels) or lights-off (Fig 4B–4D; right panels) and compared these to their respective BL_avgs_. Consistent with what is clear to the eye from Fig 4A, the most robust effects are seen on W2 and W3 which include increased Wake bouts during lights on and off and decreased Wake bout durations during lights off (Fig 4D). This is demonstrated with significant Friedman’s tests for counts and durations (Wake bout counts, lights on: Q = 49.17, p<0.0001, Fig 4D, left; lights off: Q = 49.52; p<0.0001, Fig 4D, right; Wake bout duration, lights off: Q = 48.94, p<0.0001, Fig 4D, left) During lights on, the number of Wake bouts increases compared to BL_avg_, but the bout length does not change. Since the number of NREM and REM bouts do not decrease during lights-on, the increase in Wake bouts is enabled by the decrease in NREM and REM bout length (B, C, left panels). Friedman’s tests show that NREM and REM bout lengths decrease: for NREM and REM during lights on (NREM, Q = 58.45; p<0.0001 Fig 4B, left; REM, Q = 30.82, p<0.0001, Fig 4C, left) Interestingly, increased Wake bout number ends on W4, yet NREM bout durations remain significantly decreased until W6. During lights off, the mean number of Wake bouts dramatically increases from 56.61 (±2.89 SEM) at BL_avg_, Fig 4C to 107.91 (±8.17 SEM) on W2 and 85.64 (±4.99 SEM) on W3, with concurrent decreases in bout length from 556.80s (±31.61 SEM) at BL_avg_ to 189.59s (±14.75SEM) on W2 and 277.99s (±26.62SEM) on W3. The increased bouts and decreased bout durations for Wake staging during lights off are accompanied by significant increases in the number of NREM and REM bouts, as shown by significant Friedman’s tests (NREM, lights off: Q = 47.03; p<0.0001,; p<0.005, Fig 4B, right REM, lights off: Q = 24.20; p = 0.0010, Fig 4C, right). To summarize, oxycodone withdrawal results in profound arousal/sleep state switching and decreased bout durations. These changes suggest both severe disruption of normal rat sleep architecture during early oxycodone withdrawal and differential effects on the neural circuitry that regulates sleep/wake initiation maintenance.

To control for the possibility that oxycodone withdrawal-induced changes to sleep/wake bout architecture are modulated by the acute naloxone injections on W1, we conducted 2-way ANOVAs with repeated measures on withdrawal day (W2-W8) for NREM bout # and bout duration in naloxone vs saline rats. There were no Treatment x Day interactions or main effects of Treatment for any measurement (S1B), indicating no effect of W1 naloxone injection on NREM sleep architecture.

#### Spectral power and aperiodic EEG aperiodic slope are differentially and temporally altered during spontaneous oxycodone withdrawal

Beyond sleep architecture and diurnal rhythms, it is vital to understand if, and how, spontaneous oxycodone withdrawal affects the magnitude, directionality, and temporal dynamics of functional network activity underlying sleep. Accordingly, we examined EEG spectral dynamics for NREM, REM, and Wake across baseline B4-B6 and withdrawal W2-W8 days. We first derived estimates of relative spectral power for the canonical frequency bands delta (1–4 Hz), theta (4–8 Hz), alpha (8–12 Hz), beta (12–30 Hz), and gamma (30–50 Hz). Spontaneous oxycodone withdrawal had the most robust effects on spectral power and aperiodic 1/f slopes during NREM. As such, our results and discussion focus on NREM, although data from REM and Wake is presented in the supplementary data in S1 File.

**Fig 5:**
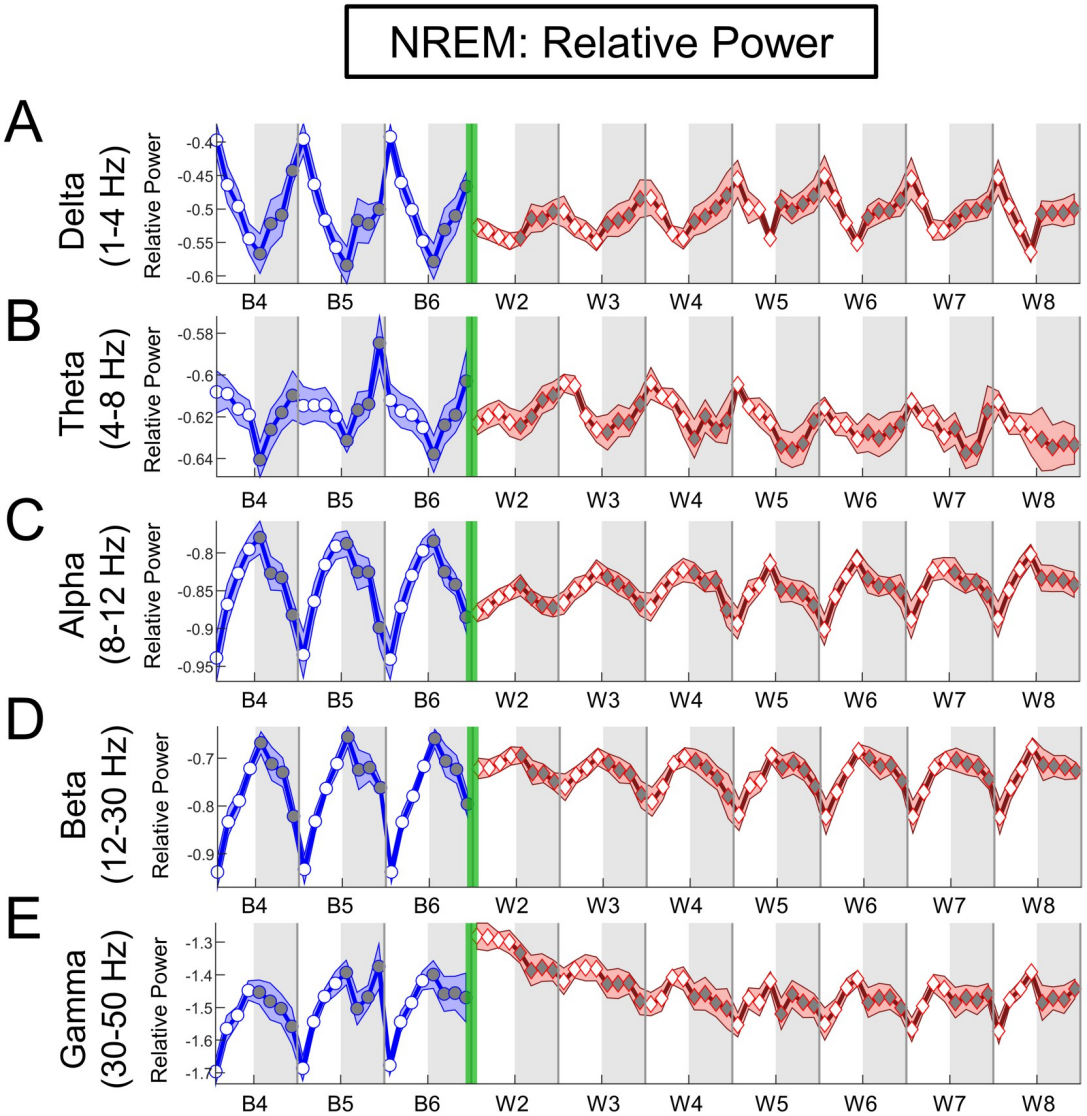
Spontaneous oxycodone withdrawal transiently eliminates diurnal changes in NREM depth. The mean relative power for NREM is plotted in 3-h bins (±SEM) for (**A**) Delta (1-4Hz), (**B**) Theta (4-8Hz), (**C**) Alpha (8-12Hz), (**D**) Beta (12-30Hz) and (**E**) Gamma (30-50Hz) frequency bands throughout baseline days 4, 5, and 6 (B4, B5, B6; blue line, circles) and spontaneous withdrawal days 2–8 (W2-W8; red line, diamonds). Vertical green bars between B6 and W2 represent the 14-d escalating oxycodone dose regimen and W1. Grey shaded regions represent ZT12-24 (lights off), and unshaded regions represent ZT0-12 (lights on). Oxycodone withdrawal has a global effect on relative power, rather than modulating specific frequency ranges. N = 8 rats. Underlying data is in Fig5_Data in S1 File.

*Spontaneous oxycodone withdrawal transiently flattens the diurnal rhythmicity of NREM relative spectral power*, *but not that of REM or Wake*. Given the flattening of baseline diurnal rhythms within sleep architecture, we next analyzed EEG relative spectral power to assess any effects observed concurrently during spontaneous oxycodone withdrawal. Qualitatively, NREM relative power measured over baseline (B4-B6) shows a clear rhythmicity [52] for each canonical frequency band (Fig 5), with the exception of theta (Fig 5B). For delta, relative power is highest at the beginning of lights-on (ZT0), decreases until ZT12 where it is at its lowest, and increases again within ZT12-24. In contrast, alpha, beta, and gamma show the opposite pattern, with the lowest relative power occurring at ZT0. During oxycodone withdrawal, NREM rhythms of power appear to flatten before gradually recovering to baseline periodic structure by W8. To determine if the rhythmicity of power was affected by oxycodone withdrawal, we quantified the DI for NREM across all frequency bands (Fig 5A–5E; left panels). A Friedman’s test revealed significant decreases to the DI for every frequency band; including theta, which had a qualitatively unclear rhythm (Delta, Q = 34.54, p<0.0001; Fig 5A; Theta, Q = 18.21, p<0.0111; Fig 5B; Alpha, Q = 38.67, p<0.0001; Fig 5C; Beta, Q = 37.71, p<0.0001; Fig 5D; Gamma, Q = 28.88, p = 0.0002; Fig 5E). Thus, spontaneous oxycodone withdrawal flattens NREM rhythms for all frequency bands.

Qualitatively, REM and Wake relative power measured over baseline B4-B6 days appear to show subtle rhythmicity at individual frequency bands (S3, S5, Figs in S1 File, respectively), but more detailed analyses of these potential rhythms were not performed, as they are beyond the scope of this initial study. Furthermore, DI analyses did not reveal any significant differences between relative power at BL_avg_ compared to W2-W8 across frequency bands for either sleep stage (S4 Fig in S1 File, REM; S6 Fig in S1 File, Wake, left panels).

**Fig 6:**
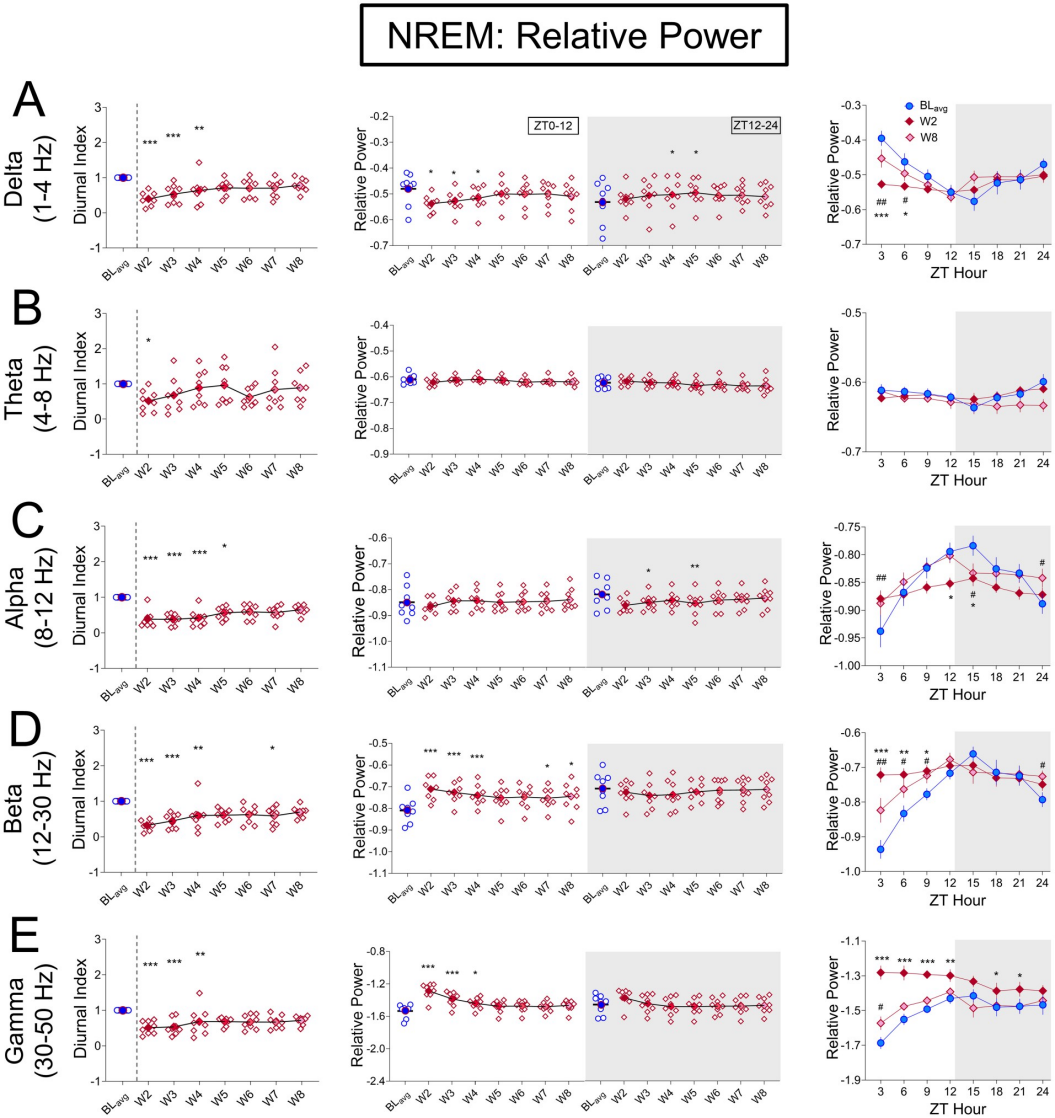
Spontaneous oxycodone withdrawal initially flattens NREM power rhythms, decreases low frequency and increases high frequency power. For relative power during NREM, the mean (±SEM) Diurnal Index (DI; **left panels**), mean (±SEM) relative power per frequency band for ZT0-12 or ZT12-24; **center panels**, and the mean (±SEM) relative power per frequency band in 3-h bins for BL_avg_, W2, and W8 (**right panels**) are shown for (**A**) Delta 1-4Hz, (**B**) Theta (4-8Hz), (**C**) Alpha (8-12Hz) (**D**) Beta (12-30Hz), (**E**) Gamma (30-50Hz) frequency bands. Non-parametric Friedman with Dunn’s post hoc tests (**left and center panels**), and two-way repeated measures ANOVA with Dunnett’s post hoc tests (**right panels**) compared W2-W8 to B_avg_. ***p<0.001.**p<0.01, *p<0.05. N = 8 rats. Underlying data is in Fig6_Data in S1 File. *Abbreviations*: ZT, zeitgeber time; BL_avg_, Baseline average; W, withdrawal.

*Early spontaneous oxycodone withdrawal has global effects on NREM relative power across frequencies*, *with minimal effects on REM and Wake*. We next quantified the mean NREM, REM, and Wake relative power for BL_avg_ and W2-W8 during either lights-on or lights-off (Fig 6, NREM; S4 Fig in S1 File, middle panels, REM; S6 Fig in S1 File, middle panels, Wake) and performed Friedman’s Tests followed by Dunn’s post hoc tests for significance. For NREM, spontaneous oxycodone withdrawal shows a continuous gradient in power changes with increasing frequency during lights-on, but sparse changes in lights-off. Compared to BL_avg_, relative power during NREM delta is significantly decreased for lights on (Q = 21.04, p = 0.00371; Fig 6A, middle white). Between 4–12 Hz (theta and alpha) there is a switch in directionality such that relative power in higher frequencies (12–50 Hz, beta and gamma) is significantly increased for lights on (beta, Q = 27.13, p = 0.0003; Fig 6D, middle white; gamma, Q = 39.67, p<0.0001; Fig 6E, middle white). During lights-off, NREM relative power shows a few significant changes compared to BL_avg_. Interestingly, these (moderate) changes peak on later withdrawal days than those observed for % time in sleep states, sleep/wake bout architecture disruption, and diurnal rhythmicity. Specifically, the peak increase in NREM relative power compared to BL_avg_ during lights-off occurs on W5 for delta (1–4 Hz; Q = 14.79, p = 0.0388; p = 0.0154 at W5, Dunn’s test, Fig 6A, middle gray panel). The strong effect of oxycodone withdrawal on NREM relative power magnitude during lights-on compared to lights-off can best be seen in the right panels of Fig 6. In particular, spontaneous oxycodone withdrawal dramatically decreases delta power and increases beta and gamma power during lights-on, with the maximum effects on W2 occurring between ZT0-9. This is demonstrated with 2-way repeated measures ANOVAs and significant 3-h bin × Treatment Day interactions (delta, F_(2.085, 14.60)_ = 9.130, p = 0.0025, Fig 6A, right panel; beta, F_(1.838, 12.87)_ = 11.92, p = 0.0014, Fig 6D, right panel; gamma, F_(3.081, 21.57)_ = 13.66, p<0.0001, Fig 6E, right panel). This suggests that oxycodone withdrawal has long-lasting effects on oscillatory activity when transitioning into the sleep period (lights-on). Across W2-W8, oxycodone withdrawal did not have robust or consistent effects on REM or Wake power during lights-on/off, as analyzed and shown in S4 Fig in S1 File, REM and S6 Fig in S1 File, Wake. A finding shared by all three sleep stages is that relative power is at BL_avg_ levels by W8 across all frequency bands, broadly similar to % time in sleep stages and sleep/wake bout architecture (Figs 2 and 4).

To control for the possibility that oxycodone withdrawal-induced changes to NREM power are modulated by the acute naloxone injections on W1, we conducted 2-way ANOVAs with repeated measures on treatment day (BL_avg_, W2, and W8) for NREM relative power at Delta and Gamma frequency ranges in 3-h bins in naloxone vs saline rats. For Gamma, we found significant Treatment x Day interactions: Gamma: F_(21,84)_ = 2.473, p = 0.0019 (S1C Fig in S1 File). However, Bonferroni’s posthoc tests showed no significant pairwise effects between naloxone and saline treated rats, indicating no effect of W1 naloxone injection on subsequent spontaneous oxycodone withdrawal effects on this endpoint.

**Fig 7:**
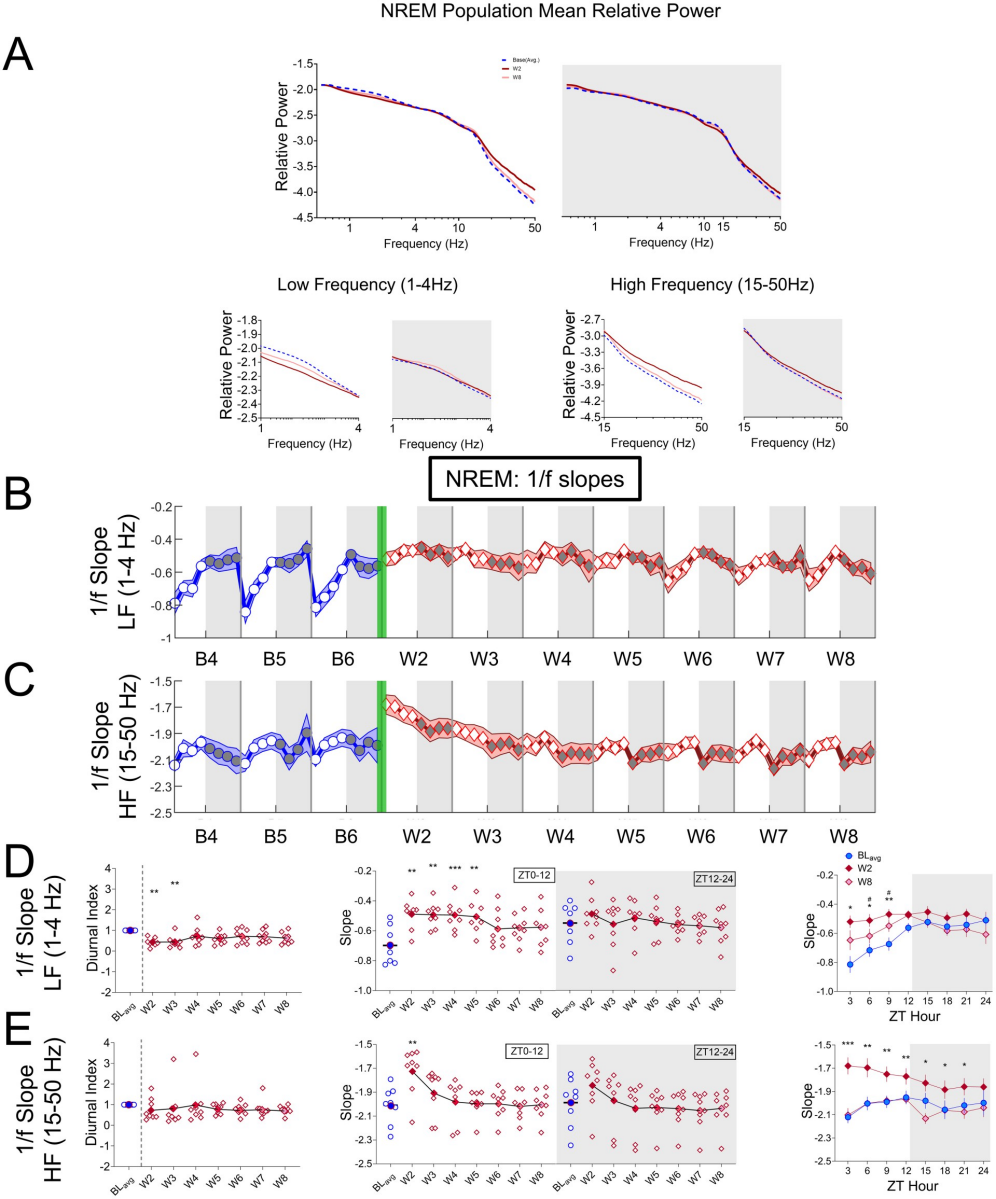
NREM 1/f slopes calculated from Power Spectrum Densities (PSDs) have a robust nocturnal rhythm at low frequencies that are initially flattened during spontaneous oxycodone withdrawal. (**A, top panel**) The average NREM PSD normalized to each rat’s total power from 0.5-50Hz plotted on a log-log scale for lights-on (ZT0-12 unshaded) and lights-off (ZT12-24; shaded) for BL_avg_ (blue), W2 (red), and W8 (pink). (**A, bottom panels**) Zoomed-in low frequency (LF, 1–4 Hz) and high frequency (HF, 15–50 Hz) portions of the NREM PSD during lights-on and lights-off are plotted and used to calculate LF and HF 1/f slopes. The mean NREM 1/f slope (±SEM) per 3-h bin is plotted for (**B)** low frequency and (**C**) high frequency ranges across baseline days 4, 5, and 6 (B4, B5, B6; blue line, circles) and spontaneous withdrawal days 2–8 (W2-W8; red line, diamonds). Vertical green bars between B6 and W2 represent the 14-d escalating oxycodone regimen and W1. For both LF (**D**) and HF (**E**) NREM 1/f slopes, the mean (±SEM; including individual rat values) Diurnal Index (**left panels**), 1/f slopes (ZT0-12 or ZT12-24; **center panels**), and 3-h bin data for BL_avg_, W2, and W8 (**right panels**) are shown. Non-parametric Friedman tests with Dunn’s post hoc tests (**left and center panels**), and two-way repeated measures ANOVA with Dunnett’s post hoc tests (**right panels**) compared W2-W8 to B_avg_. ***p<0.001.**p<0.01, *p<0.05. N = 8 rats. Underlying data is in Fig7_Data in S1 File. *Abbreviations*: ZT, zeitgeber time; BL_avg_, Baseline average; W, withdrawal.

*Spontaneous oxycodone withdrawal transiently flattens NREM aperiodic slopes at low frequencies*, *with minimal effects on REM and Wake*. The gradient of spectral power changes observed in lights-on NREM during spontaneous withdrawal raises the possibility that the entire spectrum is uniformly affected rather than a series of variable band-specific changes. Specifically, these changes are consistent with a generalized “tilt” in the aperiodic spectrum around theta/alpha ranges. We therefore explicitly assessed this possibility by quantifying the aperiodic piece-wise low frequency (LF, 1-4Hz) and high frequency (HF, 15-50Hz) spectral slope and their changes during the experiment. For visualization, we show example NREM relative spectra (in log-log) taken from a rat at baseline (BL_avg_), W2, and W8 during lights-on (ZT0-12) and during lights-off (ZT12-24) (Fig 7A, top panels) with a close-up of the HF and LF regions (Fig 7A, lower panels). For the LF lights-on (lower, left), we observe a distinct flattening of the spectral slope at W2 (red), pivoting down from a point at ~4Hz. For the HF-lights on and lights off (lower, right), we also see a flattening in the W2 (red) slope, but this time pivoting up from the lower end at ~15 Hz. To support the validity of separately analyzing LF and HF portions of the PSD to determine spectral slopes, we calculated 1/f slopes for NREM PSDs at 3 LF frequency ranges (1–4 Hz, 1–10 Hz, and 1–15 Hz; S7A-S7C Fig in S1 File) and at 3 HF frequency ranges (15–50 Hz, 15–30 Hz, and 15–45 Hz; S8A-S8C Fig in S1 File). For both the LF and HF ranges, the DI, 1/f slopes, and 3-h bin data for BL, W2, and W8 were respectively similar, suggesting a general robustness of our findings to the aperiodic frequency range.

To characterize aperiodic spectral dynamics for baseline and withdrawal days, we computed LF and HF slopes in 3-h bins for each sleep stage (Fig 7B and 7C, NREM; S9A, S9B Fig in S1 File, REM; S10A, S10B Fig in S1 File, Wake). As with relative power, we found that the most robust effects of oxycodone withdrawal on aperiodic activity occurred during NREM. Interestingly, the LF aperiodic signal (Fig 7B, blue) has a robust, diurnal rhythm at baseline that is similar in shape and directionality to the NREM periodic alpha, beta and gamma rhythms (Fig 5C–5E). To quantify rhythmicity changes to aperiodic slope at either LF or HF, we calculated the DI across all sleep stages by comparing W2-8 to BL_avg_ (Fig 7D and 7E, NREM; S9C, S9D Fig in S1 File, REM; S10C, S10D Fig in S1 File, Wake; left panels). For NREM, oxycodone withdrawal significantly decreases the LF slope DIs (Friedman’s test: Q = 22.46, p = 0.0021, Fig 7D, left). HF slope DIs did not change during withdrawal for NREM (Fig 7E, left). REM and Wake slopes appear highly variable and complex (S9, S10 Figs in S1 File), and quantification of slope DIs also revealed no significant differences compared to BL_avg_ for Wake (S10C, S10D Fig in S1 File, Wake, Figs, left panels). although there are some differences in DIs for HF REM slopes (Friedman’s test, Q = 27.92, p = 0.0002, S9D Fig in S1 File, REM, left panel).

To quantify the daily changes in slopes during withdrawal, we plotted NREM, REM, and Wake LF and HF slopes for BL_avg_ and W2-W8 separately for lights-on and lights-off (Fig 7D and 7E, NREM; S9C, S9D Fig in S1 File, REM; S10C, S10D Fig in S1 File, Wake; middle panels). We found significantly different slope magnitudes during withdrawal for all sleep stages, with the greatest effects in NREM. For NREM, the LF slope is significantly closer to 0 (indicates the slope is becoming flatter) on W2-W5 during lights-on when compared to BL_avg_ (Q = 30.71, p<0.0001; Fig 7D, middle white) but not during lights-off. This suggests that oxycodone withdrawal prevents normal light-induced changes to LF aperiodic activity. The effects of withdrawal on lights-on sleep metrics are the most prolonged for NREM. During withdrawal, NREM HF 1/f slopes are also significantly flattened (approaching 0) during lights on (Q = 29.88 p = 0.0001; Fig 7E, middle white), but this effect is only on W2. For REM, slopes are significantly different compared to BL_avg_ during lights-on and lights-off for LF (lights-on: Q = 14.71, p = 0.0399; S9C Fig in S1 File, middle white; lights-off: Q = 24.50, p = 0.009; S9C Fig in S1 File, middle gray), and during lights on for HF (lights on: Q = 23.92; p = 0.0002, S9D Fig in S1 File, middle white). Slopes at LF become steeper, unlike those for NREM. We report these findings, however given the low percent time in REM, additional data collection or experiments targeting REM-specific pathways may be required confirm these results.

Aperiodic activity follows the same patterns as those of power, sleep/wake %, activity, and temperature wherein oxycodone withdrawal effects are strongest at W2 before gradually returning to BL_avg_ levels by W8. Despite absolute (ZT0-24), or lights-on (ZT0-12) and lights-off (ZT12-24) levels returning to normal, we have repeatedly observed time-of-day effects on W8. This suggests that certain structural changes to behavior are longer lasting than others, and recovery from oxycodone withdrawal may not be complete by W8. As such, we determined the magnitude and time-of-day dependence for slopes at each sleep stage on BL_avg_, W2, and W8 in 3-h bins (Fig 7D and 7E, NREM; S9C, S9D Fig in S1 File, REM; S10C, S10D Fig in S1 File, Wake; right panels). For NREM, HF slopes are significantly flattened during both lights-on and lights-off on W2 (Fig 7E, middle panels). When W2 slopes are calculated for 3-h bins, a more detailed picture emerges in which HF slopes are significantly increased for all but the ZT21-24 bin (Fig 7E, right panel), yet the most robust flattening of slopes clearly occurs at the beginning of lights-on. A two-way ANOVA reveals a 3-h bin × Treatment Day interaction (HF: F_(3.745, 26.21)_ = 5.667, p = 0.0024), with Dunnett’s post hoc tests demonstrating significant pairwise differences in 1/f slopes between BL_avg_ and W2 for each time point except ZT21-24. For REM, there is a significant steepening of lights on/off slopes (slopes become more negative) beginning at W3 for LF EEG signals (S9C Fig in S1 File, middle panel), but we noted that quantitatively, the effect sizes are quite small. NREM HF slopes recover to baseline values by W8 in the 12-hour bin analyses. However, the more granular 3h analysis still shows significant differences in NREM LF slopes on W8 from ZT3-9 (Fig 7D, right panel). This suggests that oxycodone withdrawal may still have transient long-lasting effects.

#### Chronic saline does not change sleep-wake rhythms

For all rats, we used a within-subjects design in which telemetry data collected after cessation of drug infusions (i.e., withdrawal data) for each rat are compared to that rat’s average baseline data (BL_avg_). However, this design does not allow direct detection of any deterioration of EEG/EMG signal over time or possible effects of rat age, weight, and general health on EEG/EMG signal or sleep stage architecture and dynamics. To control for these possibilities, 4 rats received 14 days of saline instead of oxycodone, and sleep data from W2, W4, W6, and W8 were analyzed. Because there are only 4 saline rats (and only 3 used for EEG dynamics analyses), experimental power for quantitative analysis is low. However, qualitative plotting of 3-h bin saline data for % time in NREM, NREM relative power and NREM LF/HF 1/f slopes across B4-B6 and W2, W4, W6, and W8 demonstrates a clear maintenance of the magnitude and temporal rhythmicity of sleep architecture and dynamics throughout the experiment (S2 Fig in S1 File).

Together, these findings under control conditions indicate that the telemetry devices maintain reliable and consistent EEG/EMG recordings for at least the length of our experiments, and that the observed sleep disruptions measured during W2-W8 of spontaneous oxycodone withdrawal can be specifically attributed to oxycodone withdrawal itself.

## Discussion

In this study, we provide a comprehensive analysis of time-varying spontaneous oxycodone withdrawal effects on the diurnal composition of discrete sleep stages and the dynamic spectral properties of the electroencephalogram (EEG) signal in male rats. Upon cessation of a 14-d non-contingent regimen of escalating-dose oxycodone, rats show a profound loss of pre-oxycodone (baseline) diurnal rhythmicity of sleep/wake architecture (% time in NREM, REM, and Wake), temperature, and activity typically seen in nocturnal mammals. In addition, diurnal rhythmicity of relative spectral band power (i.e., delta, alpha, beta, and gamma) and LF aperiodic slope during NREM is dampened. In general, these effects peak 2–3 days into withdrawal and show signs of recovery by Day 8, although there are lingering effects that extend beyond the 8-day test period. In the first several days of spontaneous oxycodone withdrawal, rats spend less time asleep (NREM, REM) and more time awake during their typical inactive/sleep period (i.e., lights-on, ZT0-12) and more time asleep/less time awake during their typical active/wake period (i.e., lights-off, ZT12-24). Both the flattening of diurnal rhythms combined with the altered sleep duration during lights on and off are consistent with early [18, 19] and more recent studies [20, 21] and with clinical observations of insomnia and daytime sleepiness in humans during opioid withdrawal and following chronic opioid use [6, 9, 10, 12]. Both sleep and wake bout architecture are disrupted during early withdrawal, suggesting widespread instability across vigilance states. Finally, detailed analyses of EEG spectral power and aperiodic slope show the greatest oxycodone withdrawal effects on NREM sleep. Relative power shows a gradient of inversely proportional change with increasing frequency, which indicates a global change in the orientation of the EEG power spectrum—thus motivating a direct study of the dynamics of the EEG aperiodic slope. For NREM, oxycodone withdrawal results in a flattening of aperiodic slopes (slope magnitude closer to 0), broadly suggesting increased neural excitation [45, 47]. These changes are consistent with a prolonged period of physiological recovery from opioid withdrawal, even as overt signs of behavioral recovery emerge (absence of classic withdrawal signs, restoration of locomotor activity). Together with the temporal dependence of sleep metrics on oxycodone withdrawal day, these foundational findings invite mechanistic studies to understand the neurobiological mechanisms engaged in opioid withdrawal’s disruption of sleep.

### Spontaneous oxycodone withdrawal disrupts the diurnal rhythmicity and architecture of sleep

As previously reported, opioids such as oxycodone have acute effects on sleep processes [22]. In our study, the ZT0-2 subcutaneous infusion of high dose oxycodone (8 mg/kg) suppresses the diurnal (baseline) increases in NREM and REM sleep and decrease in time awake that occurs when the lights turn on (ZT0). This effect lasts for ~6 h after oxycodone infusion and is thought to result from both the infusion time course and predicted brain pharmacokinetics and pharmacodynamics of oxycodone [53]. Similarly, the ZT12-14 oxycodone infusion suppresses the typical decrease in NREM and REM sleep and increase in time awake that occurs when the lights turn off (ZT12). During spontaneous withdrawal, however, sleep disruptions are robust, long-lasting, and not directly produced by drug infusion. These oxycodone withdrawal effects are the focus of this report.

The endpoints under study—NREM, REM, Wake, body temperature, and locomotor activity—all show diurnal rhythmicity under baseline conditions. For each of these endpoints—except temperature—there is a transient flattening of diurnal rhythmicity quantified using the diurnal index (DI). The reduced DI (i.e., flattening) results from a decrease in the difference between daily maximum and minimum values of behaviors during both lights-on and lights-off. Temperature (subcutaneous measure) maintains its rhythmicity, but the magnitude of the typical lights-off increase is reduced during withdrawal. This dissociation between the flattening of diurnal patterns in sleep behaviors and temperature is consistent with the finding that diurnal variation in sleep stages is dissociated from body temperature [54]. Although spontaneous oxycodone withdrawal disrupts the % of time spent in each sleep/wake stage and temperature/activity compared to BL_avg_, the most robust differences occur primarily during lights-off. Indeed, even by W8, there are significant differences in sleep/wake times during lights-off but not lights-on. Further, withdrawal-induced disruptions of temperature and activity are only observed during lights-off. Considered together, the temporal effects of spontaneous oxycodone withdrawal on daily rhythms and magnitudes of sleep/wake and temperature/activity indicate profound disruption of diurnal rhythms.

Although we did not test this directly, there is accumulating evidence in the literature that chronic opioid administration and withdrawal modulate molecular and behavioral diurnal processes [55, 56]. For example, after 30 days of abstinence in human heroin users, disruptions in the expression of PERIOD clock genes and diurnal rhythms of cortisol and endorphins persist [31]. Almost identical results were found in rats for up to 60 days of withdrawal from chronic morphine administration [30]. In studies by Li and colleagues (2010), blunted diurnal expression of PERIOD genes occurred not just in the suprachiasmatic nucleus (SCN, central pacemaker of the brain), but also in the prefrontal cortex, nucleus accumbens, and amygdala—brain regions that regulate emotional states such as reward and aversion. Behaviorally, it has been shown that long-access heroin self-administration reversed the sleep-wake cycle in rats, and during abstinence, wake and NREM returned to baseline diurnal rhythms, whereas REM sleep maintained its abnormalities for 3–6 days [57]. In addition to diurnal processes, sleep regulatory mechanisms include homeostatic processes that track sleep drive [32–35], and allostatic processes such as stress [36]. Indeed, opioid withdrawal is an extreme stressor and considered an allostatic load [36], which is consistent with reports of insomnia from people with OUD during withdrawal [11, 58]. We designed our experiments such that oxycodone administration and EEG/EMG recordings were remote and wireless, minimizing factors such as daily handling and tethering-related restrictions in free movement or posture and thereby restricting stress as much as possible to the experience of withdrawal itself.

### Reduced sleep bout durations during early oxycodone withdrawal disrupts NREM sleep depth

NREM sleep is chiefly defined by the presence of low frequency power associated with slow waves in the EEG spectrum [39]. Indeed, deep NREM is often referred to as slow wave sleep (SWS) [59], and low frequency power is considered a surrogate of sleep depth. Under normal (baseline) conditions NREM low frequency power dissipates over the course of the sleep period (ZT0-12) and is indicative of a reduction in sleep pressure [32, 58, 60, 61]. In humans, NREM sleep is most commonly defined in three stages (N1-N3), with sleep depth going from low (N1) to high (N3) [62–64]. Recently, it has been shown that slow oscillation power itself is a reliable, objective, and continuous correlate of sleep depth [65].

In rodents, however, NREM is scored as a unitary stage, and the likely existence of NREM depth in rodents has been largely ignored. This is primarily due to rodents’ comparatively short and rapid fluctuation between NREM and REM sleep, with cycles lasting around 10 min in rodents vs 1–2 hours in humans [66, 67]. Sleep spindle activity, the hallmark of N2 sleep in humans, is spectrally diffuse in rodents [68], making it harder to define, detect, and score. The convention of a single NREM state in rodents is likely more a feature of practical scoring logistics, rather than a mechanistic statement on constant sleep depth. Thus, despite lack of standardized differentiation between NREM stages, it would not be impertinent to discuss changes in low frequency power as possibly being related to depth of NREM sleep in rodents.

In these studies, we observed NREM delta power to be at its highest at ZT0 (lights on), linearly decrease from ZT0-12, and then increase again once lights are off, a pattern consistent with the dissipation of sleep pressure and changing sleep depth. Conversely, we see the opposite pattern for frequencies higher than 8 Hz, which are typically associated with wakefulness and arousal [37]. The reduction in delta power during early oxycodone withdrawal is consistent with a recent study in mice [20] showing decreased delta power during morphine withdrawal, which was hypothesized to represent a reduction in sleep pressure. However, we see a significant but small (~5%) decrease in lights-on % time in NREM during W2-W5, with a large (~20%) significant increase during lights-off for W2-W3. Consequently, the observed decrease in delta power is not likely principally driven by major reductions in sleep pressure. Rather, we observe a significant increase in the number of lights-on NREM bouts on W2 and a significant decrease in bout duration W2-W5. Specifically, lights-on NREM W2 shows a ~50% increase in the number of bouts and a ~25% decrease in bout duration, (akin to strong fragmentation of human NREM sleep) during early withdrawal. Moreover, during lights-off, there is a significant increase in the number of bouts for W2-W4, with no significant change in duration, consistent with high sleep pressure leading to extended sleep related to recovery after sleep disruption. These findings of reduced NREM bout duration and increased bout number are therefore more consistent with a model of foreshortened or fragmented NREM sleep than a reduction in sleep pressure. Future analyses can explore differences in arousals, spindle activity, and other markers of sleep depth and arousability to further elucidate potential causal mechanisms of fragmentation.

NREM fragmentation/decreased NREM bout duration has varied effects on low frequency power, as evidenced by conflicting reports describing patterns observed in narcolepsy and obstructive sleep apnea [69–71]. In the context of opioid withdrawal in humans, NREM fragmentation may increase low frequency power, and hence sleep pressure, to compensate for reduced sleep. Conversely, NREM fragmentation could also decrease low frequency power such that the subject does not have sufficient time to enter deep NREM sleep. In general, the evidence suggests that sleep fragmentation plays an important role in the changes observed during early withdrawal. Finally, it cannot be ignored that opioid withdrawal is an extreme stressor for the animal, thus providing a strong allostatic load on sleep regulation [72, 73].

Taken together, our data support the utility of viewing NREM sleep as a continuum across sleep depth (as in humans), rather than a unitary stage. However, rhythmic changes in NREM sleep depth are not the sole driver of the amount of time spent in sleep stages under baseline conditions or after oxycodone withdrawal. Diurnal effects likely play an important role: even with withdrawal-induced flattening of diurnal periodicities, clear changes in sleep metrics are consistently observed at lights-on and lights-off.

### Early withdrawal disrupts dynamics of the aperiodic component of the EEG

It is important to consider changes in the EEG spectrum as a whole, as opposed to changes in each individual frequency band as a whole. When comparing all NREM EEG power bands together during baseline with those seen during oxycodone withdrawal, the results indicate coordinated, global changes in the overall slope of the EEG spectrum with a pivot point around theta, such that low and high frequencies are anti-correlated. Thus, the within-NREM periodicity predominantly reflects cyclical changes in the aperiodic component of the EEG signal. This is confirmed by the mean NREM relative power spectra across conditions, which shows a tilt in the slope of the spectrum pivoting in the theta range on W2. This is directly quantified through direct estimation of low (1-4Hz) and high (15-50Hz) frequency (LF, HF, respectively) components of the NREM 1/f slope across the experiment. Upon withdrawal, both LF and HF components show a significant flattening (1/f slopes approach 0) for lights-on with marked loss of periodicity. Given the duration and significance of the difference, LF lights-on slope is the most sensitive indicator of oxycodone withdrawal identified in the current studies, making this endpoint a strong candidate as an EEG biomarker to track the progression of withdrawal.

Mechanistically, changes in the aperiodic slope have been hypothesized to reflect alterations in the excitatory-inhibitory balance (EI) of the brain. Indeed, pharmacological excitation of neural activity is associated with flatter slopes (i.e., slope values closer to 0), whereas inhibition is associated with steeper slopes [43, 45, 47]. Additionally, flatter 1/f slopes during NREM can also indicate a decrease in inhibitory receptor density [46, 49]. One caveat with interpreting scaling changes in 1/f slopes as shifts in EI balance is that pharmacological studies often involved compounds with multiple dose- and brain region-dependent targets [45, 46, 47]. Despite this caveat, it has been clearly demonstrated that 1/f activity is robustly modified by drugs, cognitive tasks, and psychiatric conditions [74, 75]. Our finding that spontaneous oxycodone withdrawal flattens low frequency NREM aperiodic slopes suggests that EI balance is shifted towards greater excitability in neural circuits associated with sleep. These results could also arise from differences in NREM sleep depth during withdrawal.

Studies on the molecular and electrophysiological [32–36] actions of chronic opioid exposure and withdrawal have repeatedly shown a rebound increase in neural excitation once MOR activation ceases with drug withdrawal [25, 26, 27]. The functional consequences of rebound excitation are brain region-dependent, with several MOR-expressing brain regions contained within sleep circuits [24]. As one example, morphine withdrawal increases expression of the immediate early gene, cFos, in the region of the lateral hypothalamus that produces the wake-promoting neuropeptide orexin [76]. Orexin receptor antagonists have been shown to decrease opioid withdrawal symptoms, opioid tolerance, and motivation to seek opioids in rodents, suggesting the peptide itself plays an important role in the allostatic response to chronic opioid exposure [77, 78]. Consistent with this possibility, the dual orexin receptor antagonist, suvorexant, has FDA approval for the treatment of insomnia [79] and is in clinical trials for the treatment of opioid withdrawal sleep disruptions [80].

### Limitations and future work

As is often the case with early studies. our work has important limitations that can be addressed in future studies. In particular, we used a relatively small sample size (N = 11 oxy rats, N = 4 saline rats) and only studied male rats. Future work can explore the impact of sex as a biological variable using a larger number of rats. Additionally, sleep scoring was not conducted under blinded conditions, although our approach is not uncommon in the field. The underlying EEG/EMG data are available on the National Sleep Research Resource (sleepdata.org), an NHLBI-sponsored public database of sleep data intended to advance sleep and circadian research by providing data to the public.

Indeed, future work may apply more sophisticated statistical approaches for ascertaining and quantifying the periodicity of the signals as well as for modeling the change in diurnal rhythms due to opioid withdrawal. [81, 82]. Despite these limitations, our work establishes a foundation for future studies involving subjects that have self-administered the drug, rather than receiving it by programmable mini-pump, since response contingencies can play an important role in drug-seeking behaviors that are characteristic of substance use disorders [83–85].

### Summary

In summary, these foundational studies use highly structured and objective methods to characterize the impact of oxycodone withdrawal on sleep structure and dynamics in rats. Despite the growing focus of clinical research on sleep disturbance in people receiving medication for opioid use disorder, only a small number of studies have focused specifically on opioid withdrawal and sleep [20, 21]. This represents an important gap in knowledge, as sleep disruption is both common and persistent among people receiving medication for opioid use disorder [14, 80]. The few studies examining the treatment of insomnia for opioid use disorder have shown limited efficacy for common sleep medications [86, 87] highlighting the need for improved mechanistic understanding of the impact of opioids on sleep to identify better treatment targets for alleviating sleep disruption in people with opioid use disorder. The present studies provide in-depth characterization of numerous translationally relevant sleep endpoints—including basic sleep architecture, frequency band-dependent oscillatory activity. and aperiodic EEG activity—providing the basis for future work that addresses fundamental questions about the mechanisms underlying opioid withdrawal-induced sleep deficits. This approach may facilitate the development of novel therapeutics and enable the use of sleep metrics as biomarkers to more effectively manage oxycodone withdrawal in humans.

## Supporting information

S1 File (ZIP)
