# Supplementary figures and images for: Spontaneous oxycodone withdrawal disrupts sleep, diurnal, and electrophysiological dynamics in rats
Source: PLoS One. 2025 Jan 17;20(1):e0312794. doi: 10.1371/journal.pone.0312794 (PMC11741586; doi:10.1371/journal.pone.0312794)

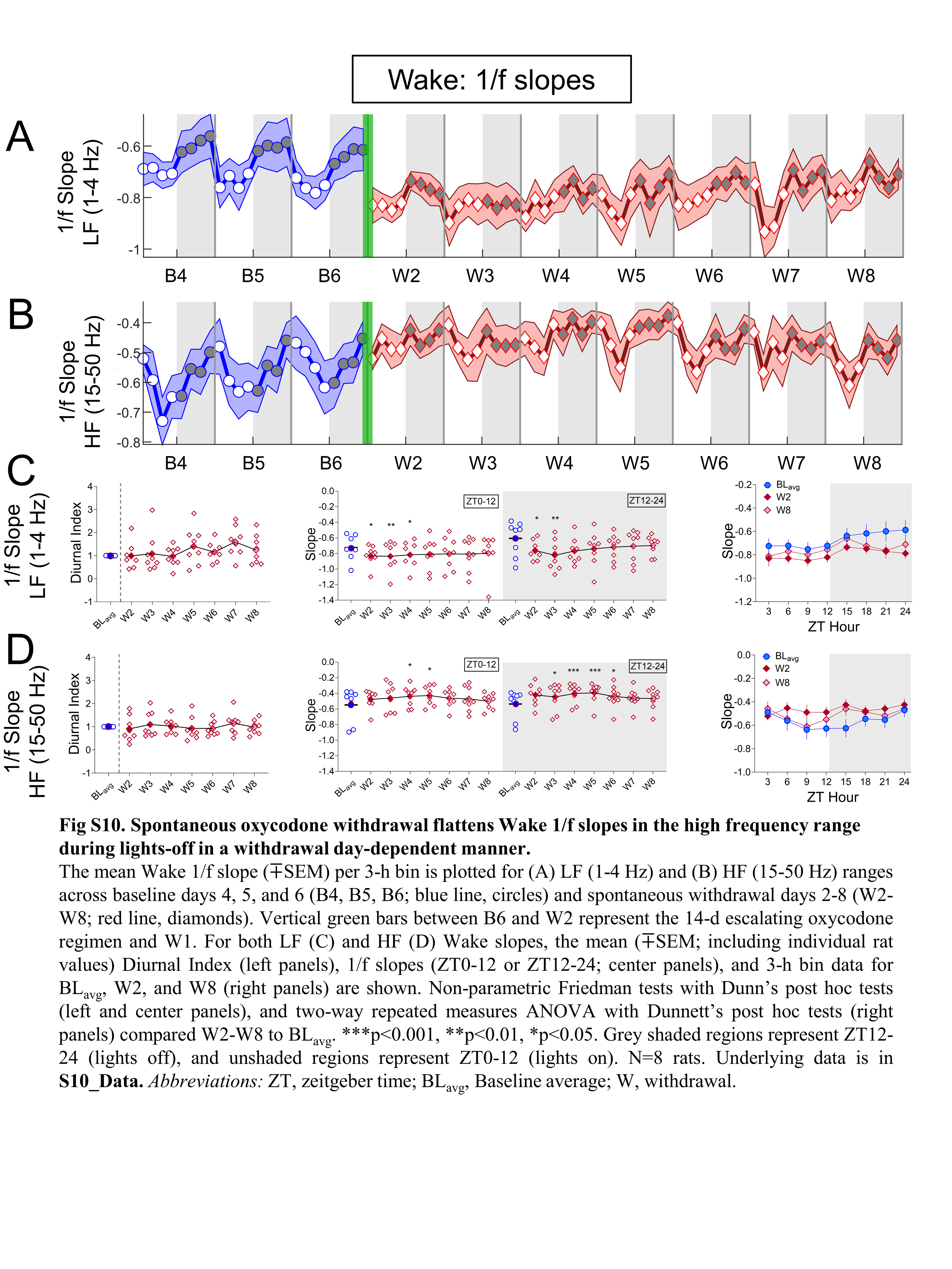

Supplement: S1 File — (ZIP) [file pone.0312794.s001.zip › All Supplementary Figures and Data 101824/S10_Fig.tif]

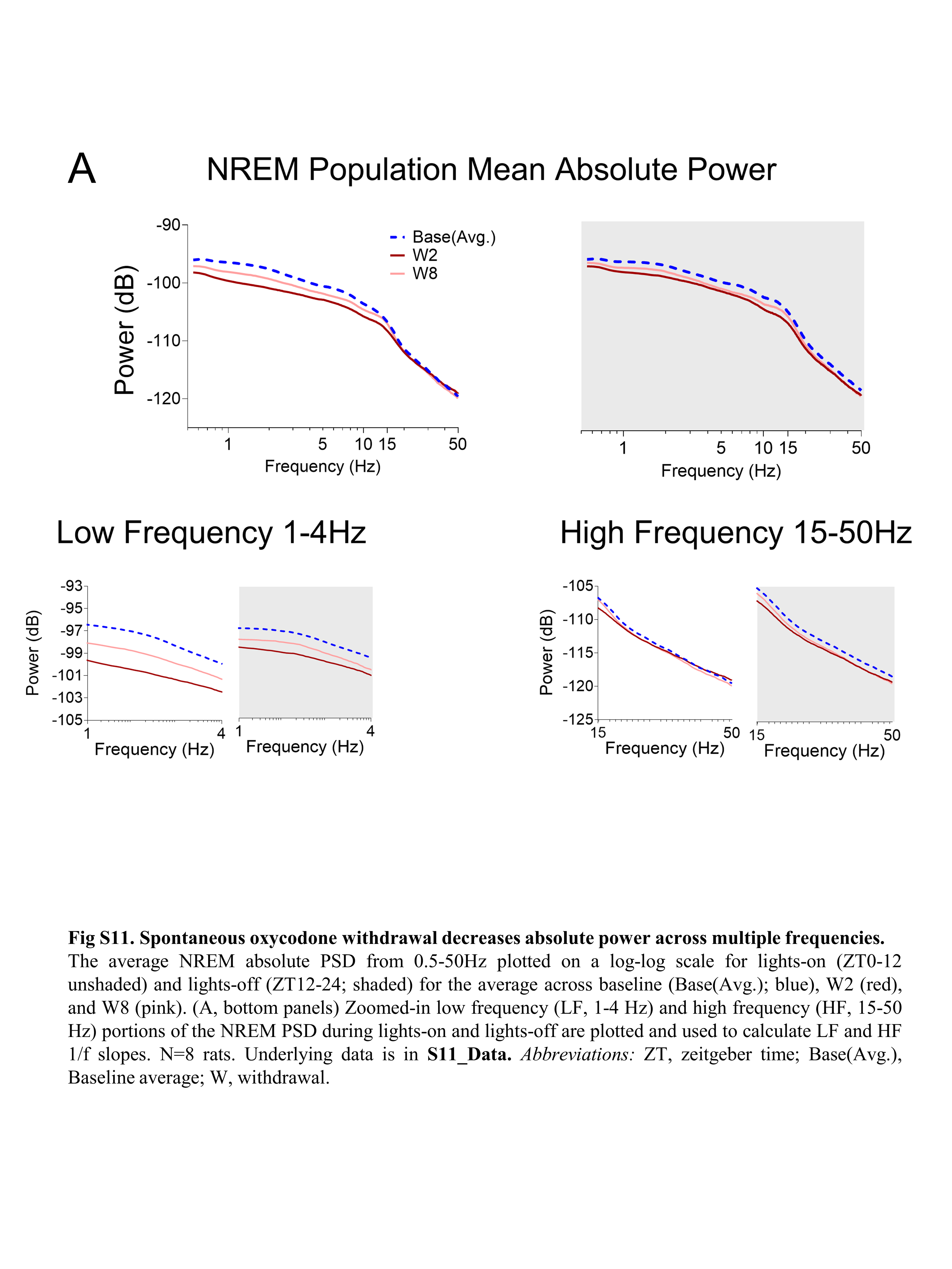

Supplement: S1 File — (ZIP) [file pone.0312794.s001.zip › All Supplementary Figures and Data 101824/S11_Fig.tif]

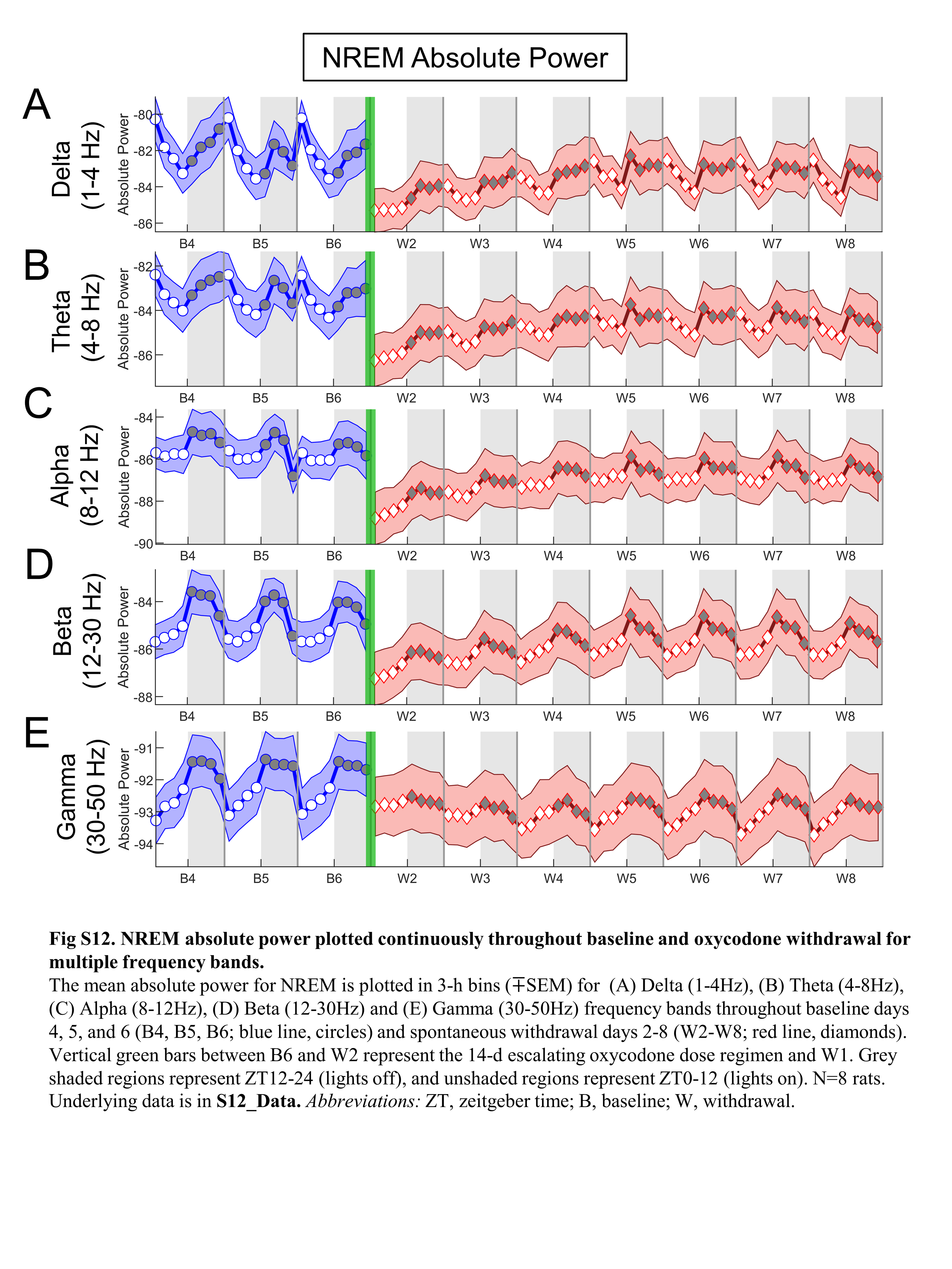

Supplement: S1 File — (ZIP) [file pone.0312794.s001.zip › All Supplementary Figures and Data 101824/S12_Fig.tif]

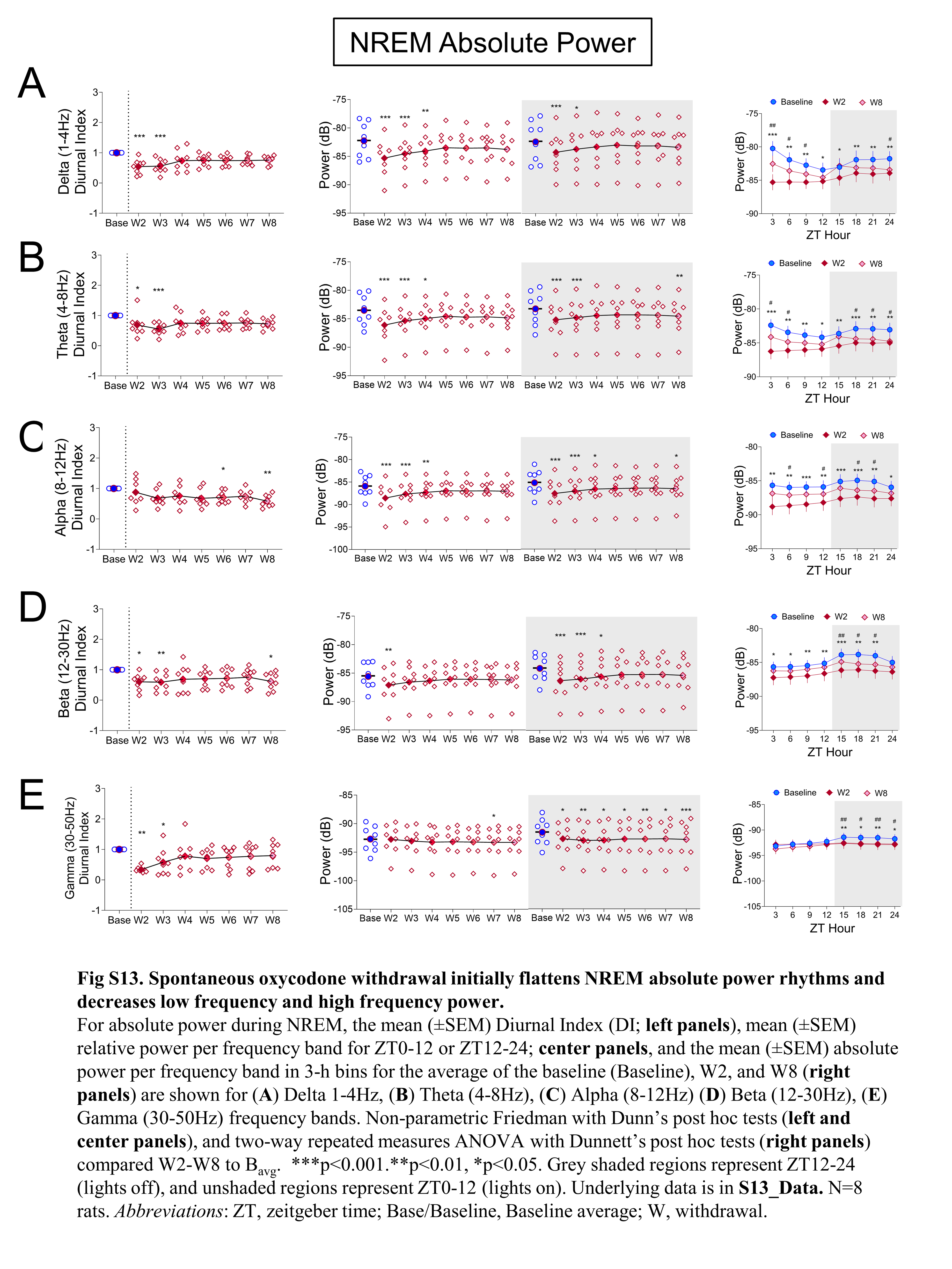

Supplement: S1 File — (ZIP) [file pone.0312794.s001.zip › All Supplementary Figures and Data 101824/S13_Fig.tif]

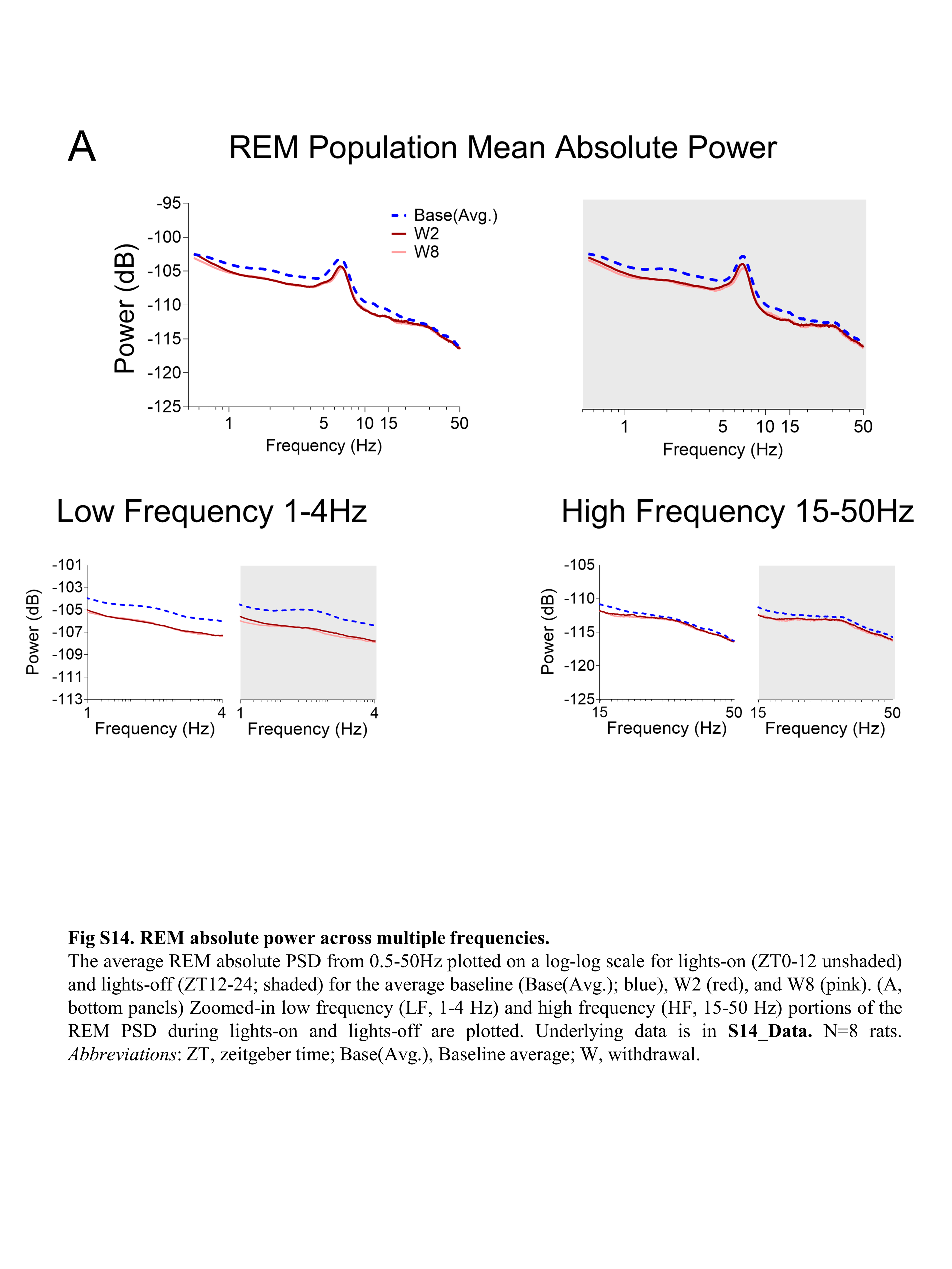

Supplement: S1 File — (ZIP) [file pone.0312794.s001.zip › All Supplementary Figures and Data 101824/S14_Fig.tif]

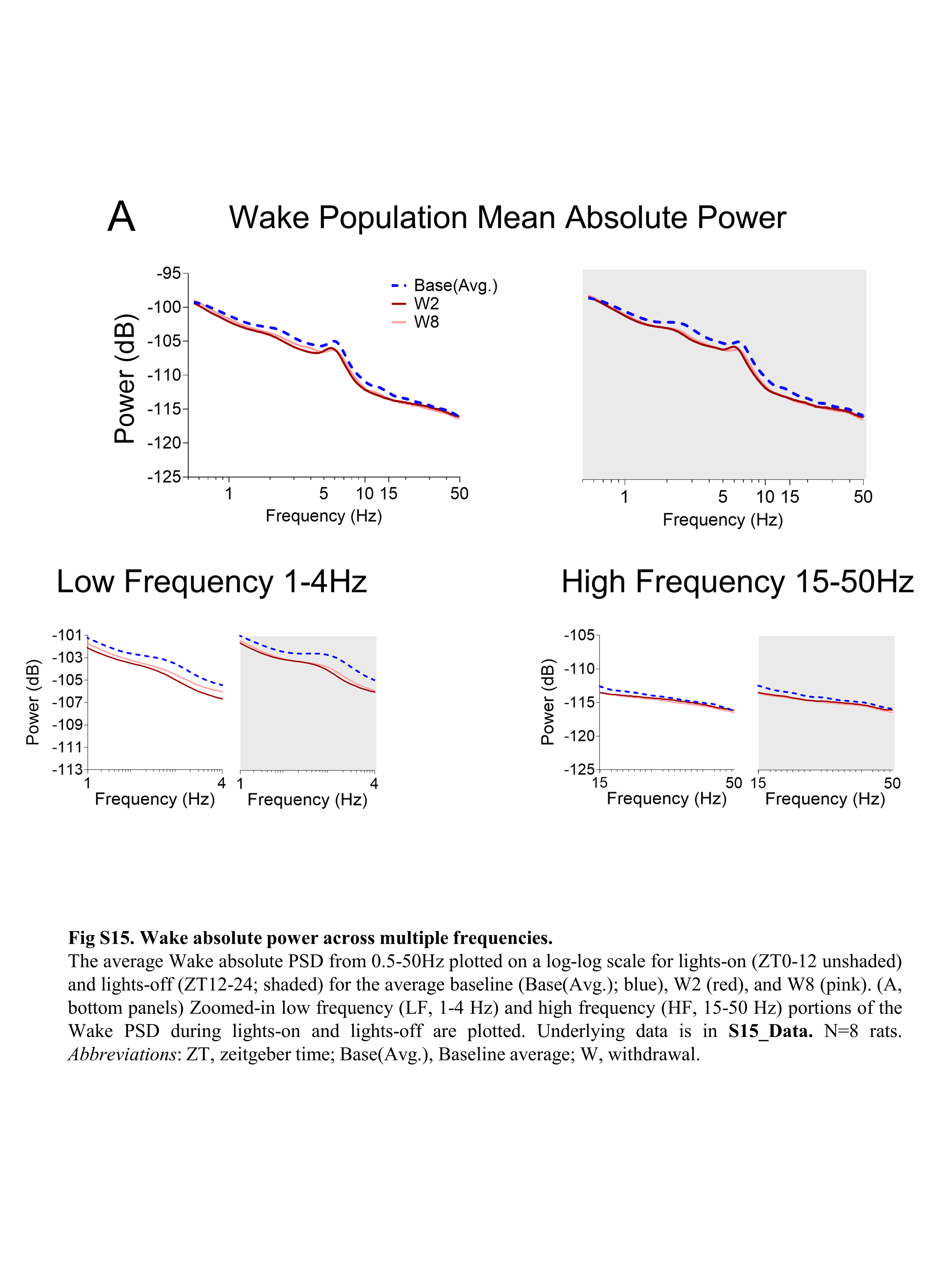

Supplement: S1 File — (ZIP) [file pone.0312794.s001.zip › All Supplementary Figures and Data 101824/S15_Fig.tif]

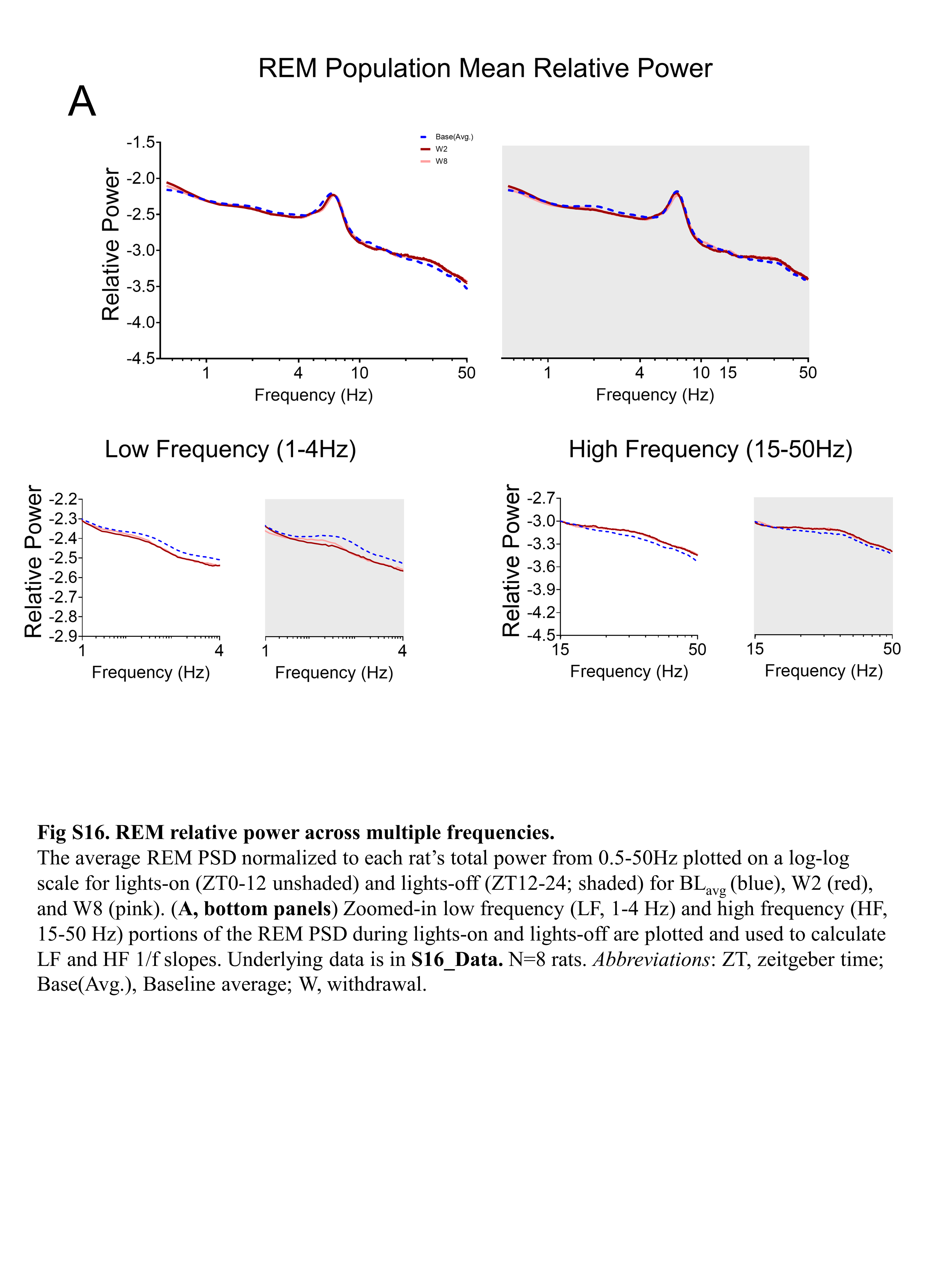

Supplement: S1 File — (ZIP) [file pone.0312794.s001.zip › All Supplementary Figures and Data 101824/S16_Fig.tif]

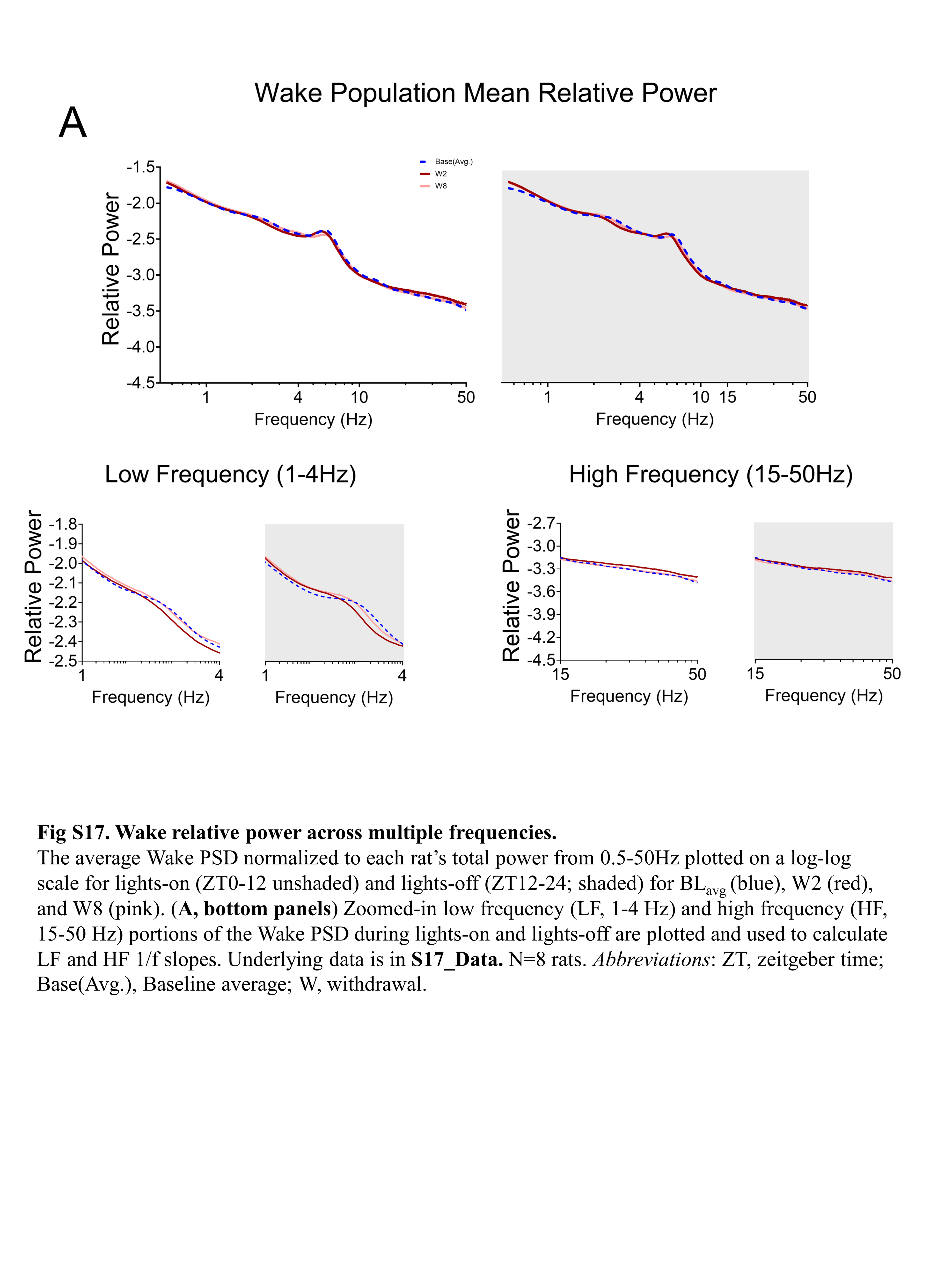

Supplement: S1 File — (ZIP) [file pone.0312794.s001.zip › All Supplementary Figures and Data 101824/S17_Fig.tif]

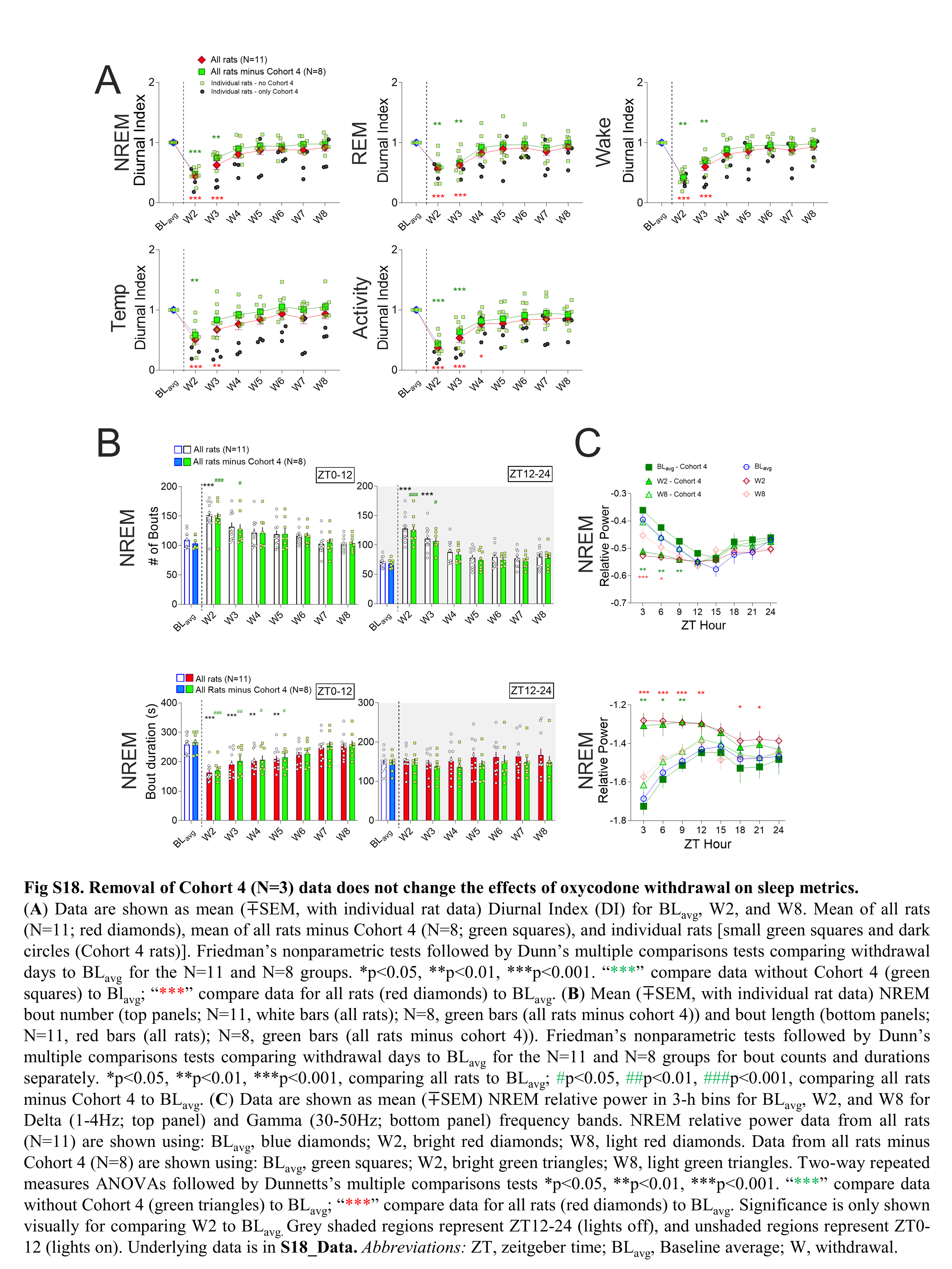

Supplement: S1 File — (ZIP) [file pone.0312794.s001.zip › All Supplementary Figures and Data 101824/S18_Fig.tif]

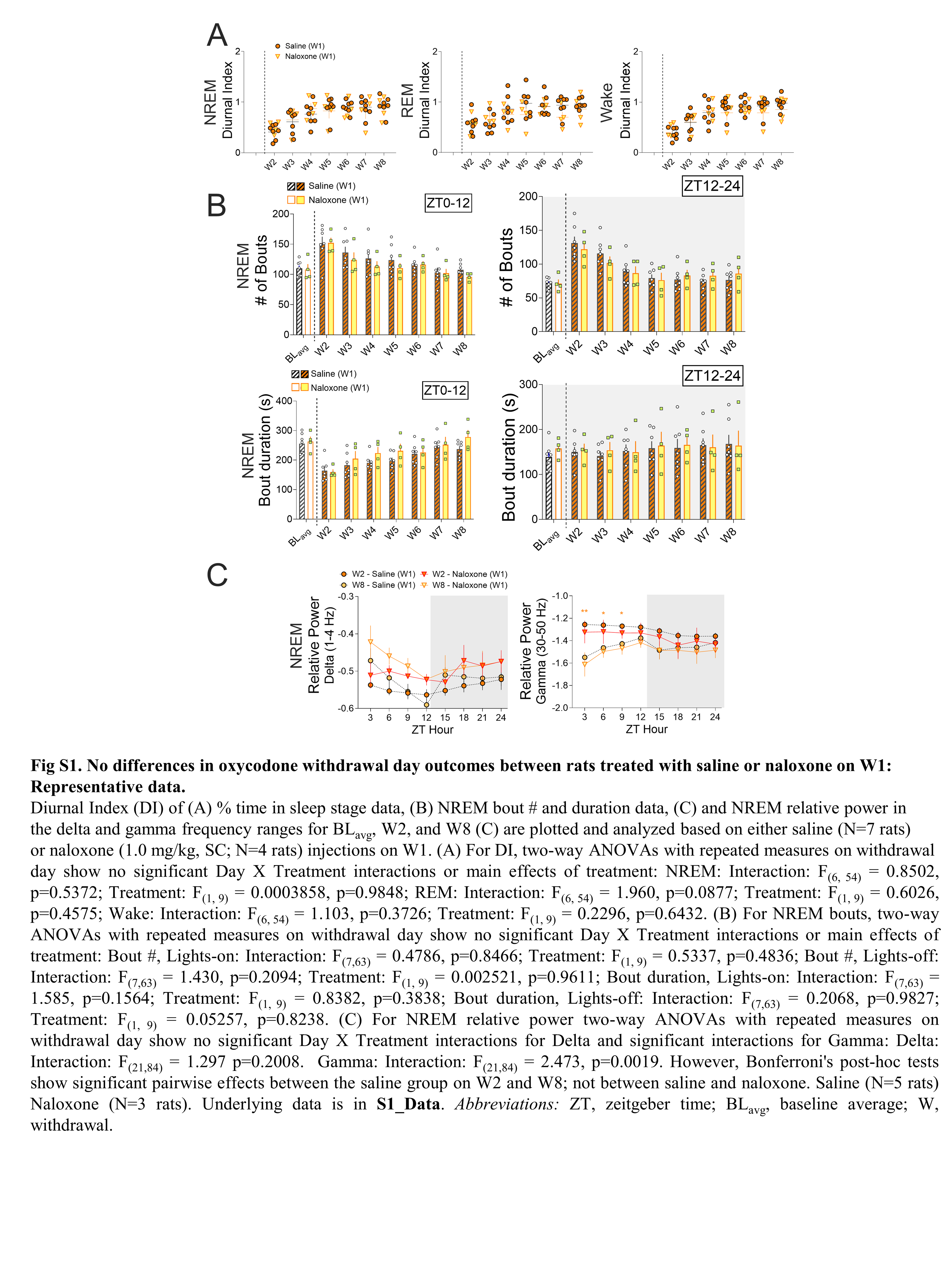

Supplement: S1 File — (ZIP) [file pone.0312794.s001.zip › All Supplementary Figures and Data 101824/S1_Fig.tif]

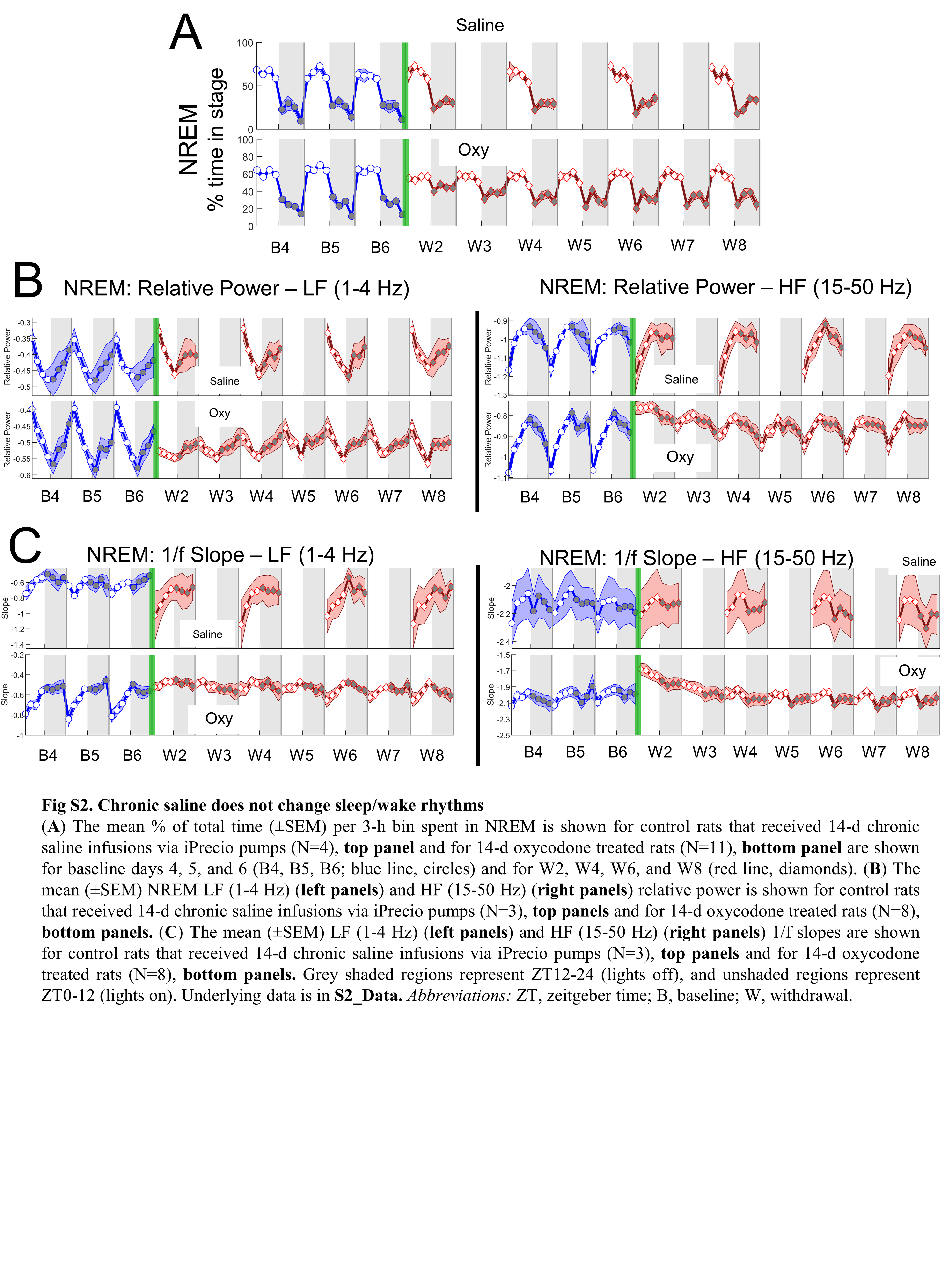

Supplement: S1 File — (ZIP) [file pone.0312794.s001.zip › All Supplementary Figures and Data 101824/S2_Fig.tif]

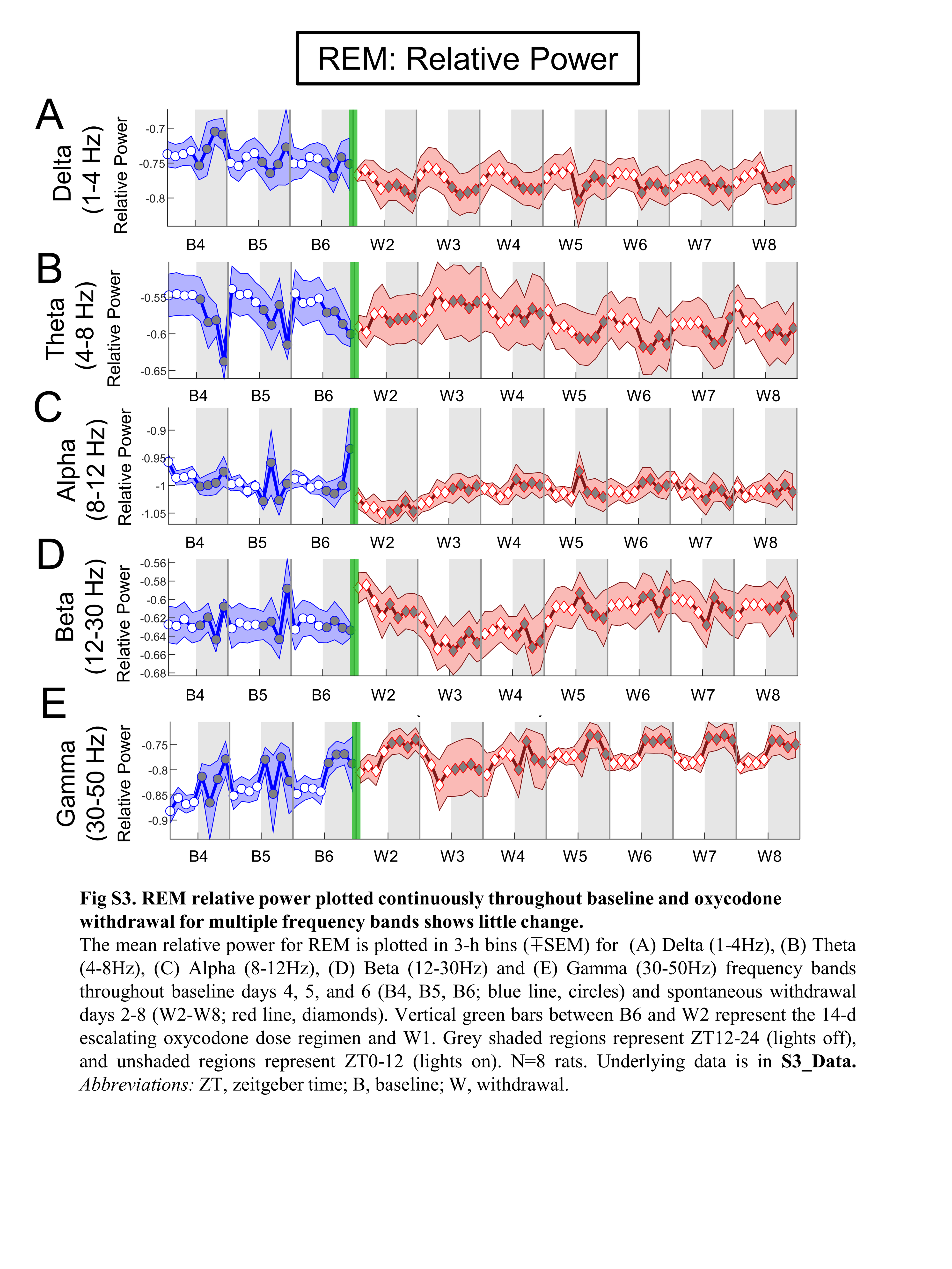

Supplement: S1 File — (ZIP) [file pone.0312794.s001.zip › All Supplementary Figures and Data 101824/S3_Fig.tif]

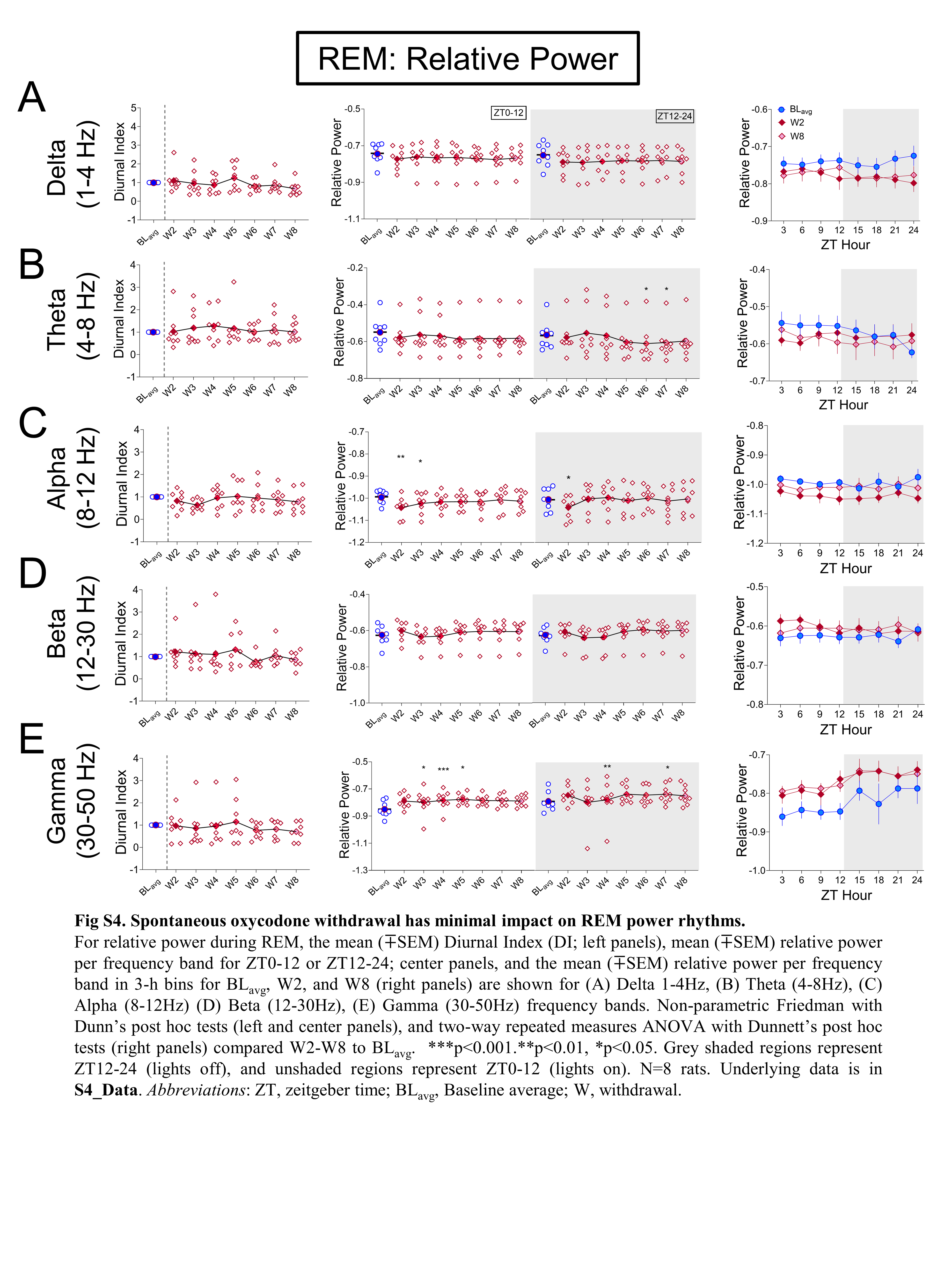

Supplement: S1 File — (ZIP) [file pone.0312794.s001.zip › All Supplementary Figures and Data 101824/S4_Fig.tif]

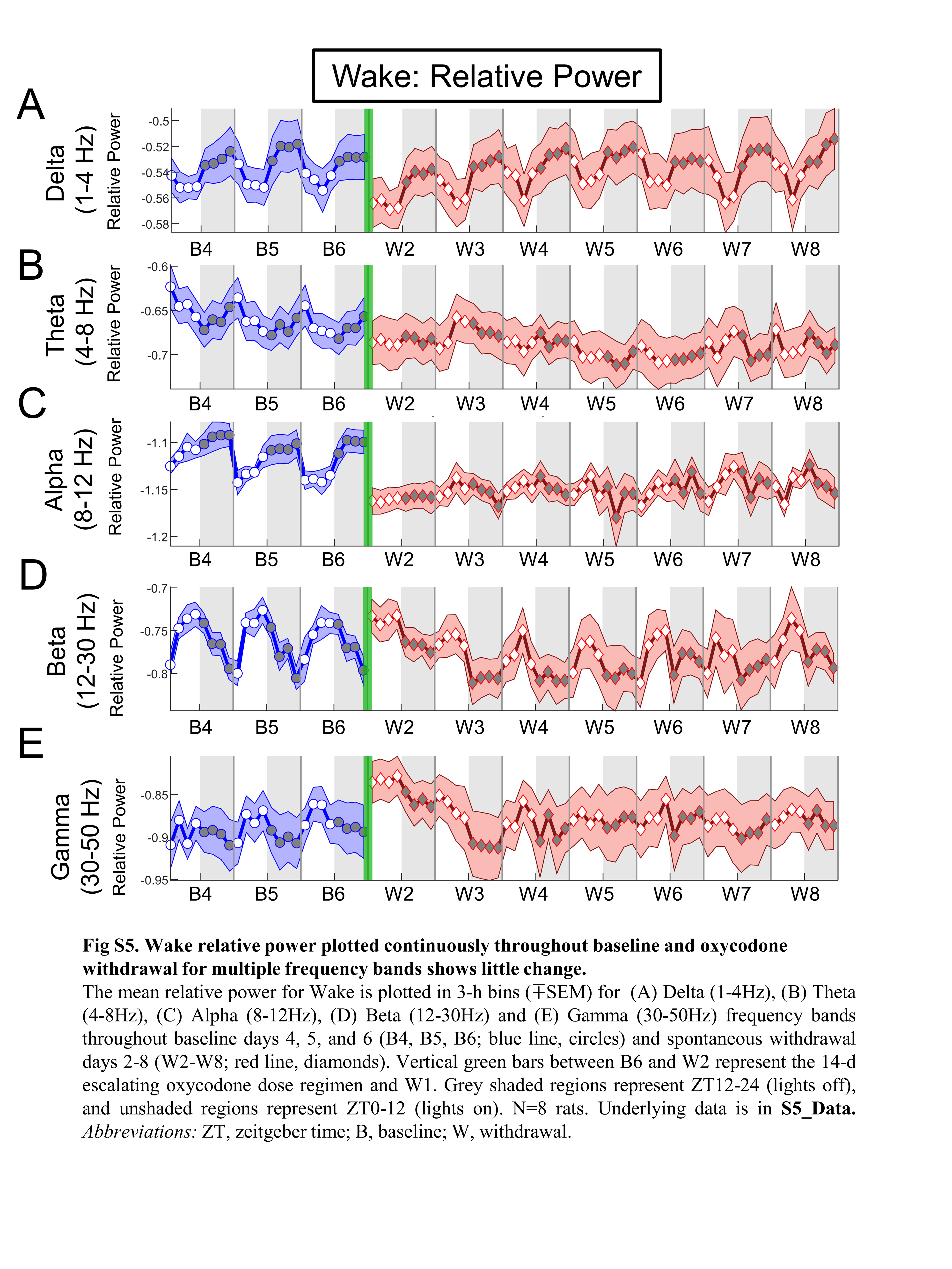

Supplement: S1 File — (ZIP) [file pone.0312794.s001.zip › All Supplementary Figures and Data 101824/S5_Fig.tif]

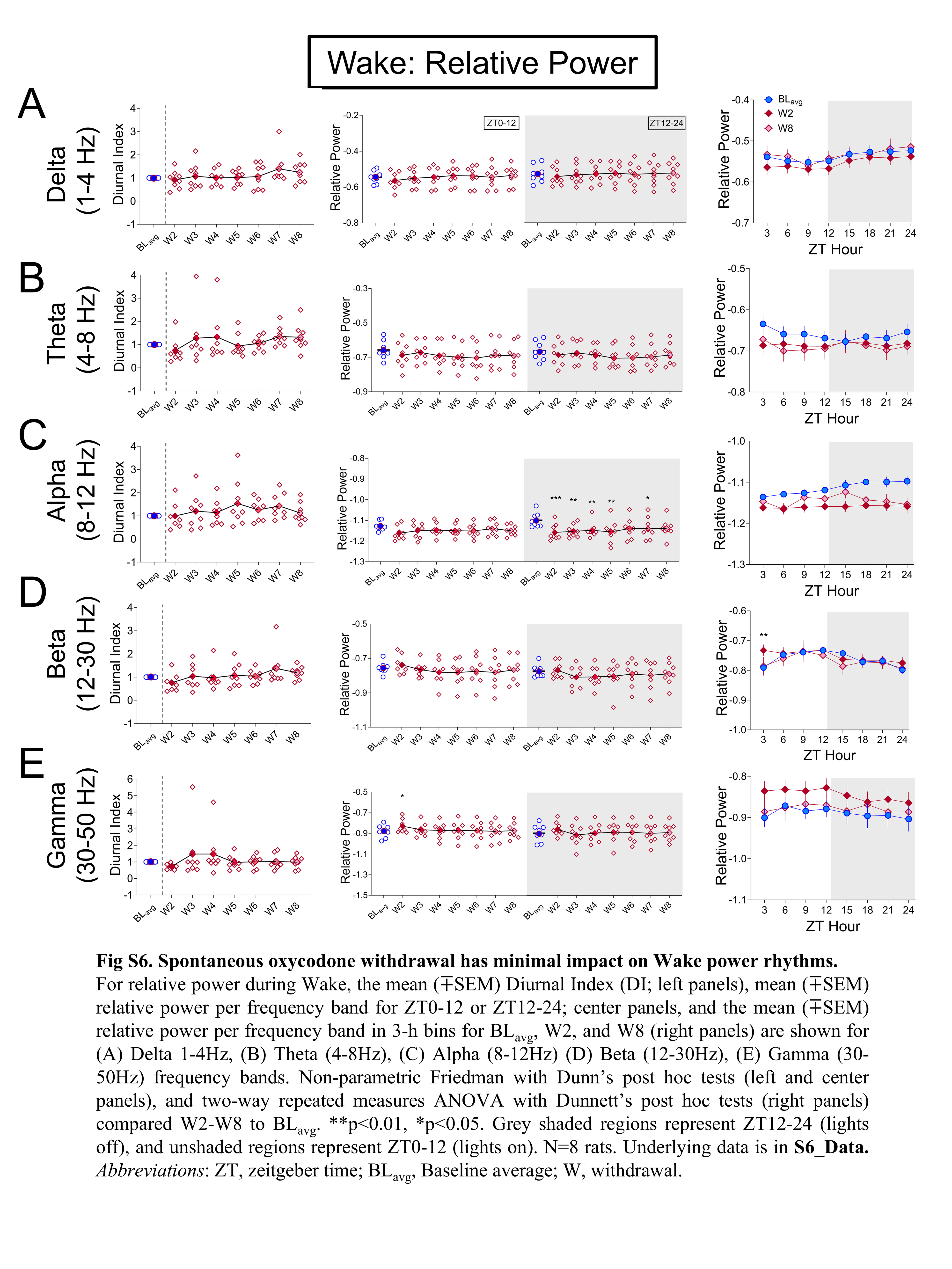

Supplement: S1 File — (ZIP) [file pone.0312794.s001.zip › All Supplementary Figures and Data 101824/S6_Fig.tif]

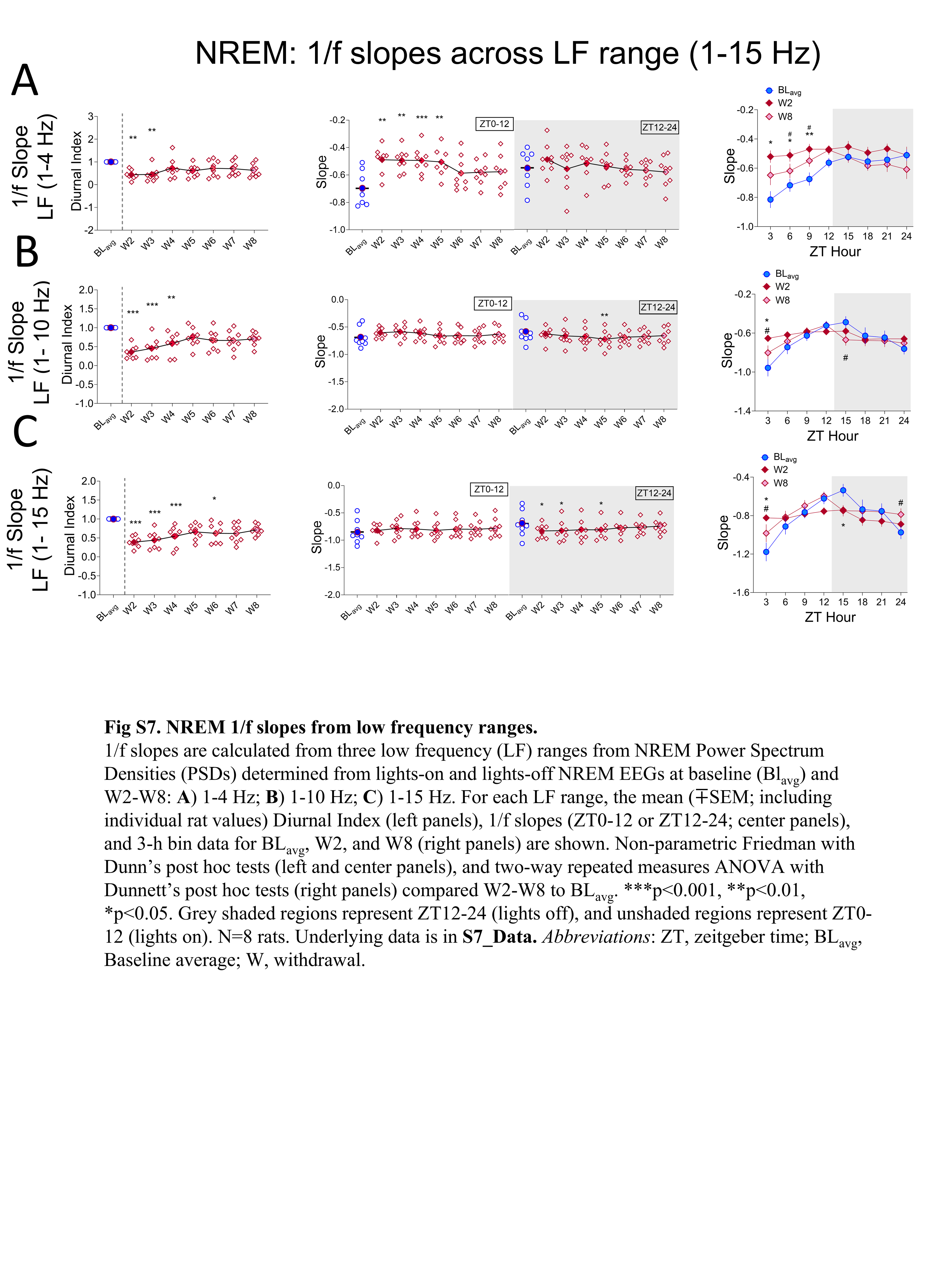

Supplement: S1 File — (ZIP) [file pone.0312794.s001.zip › All Supplementary Figures and Data 101824/S7_Fig.tif]

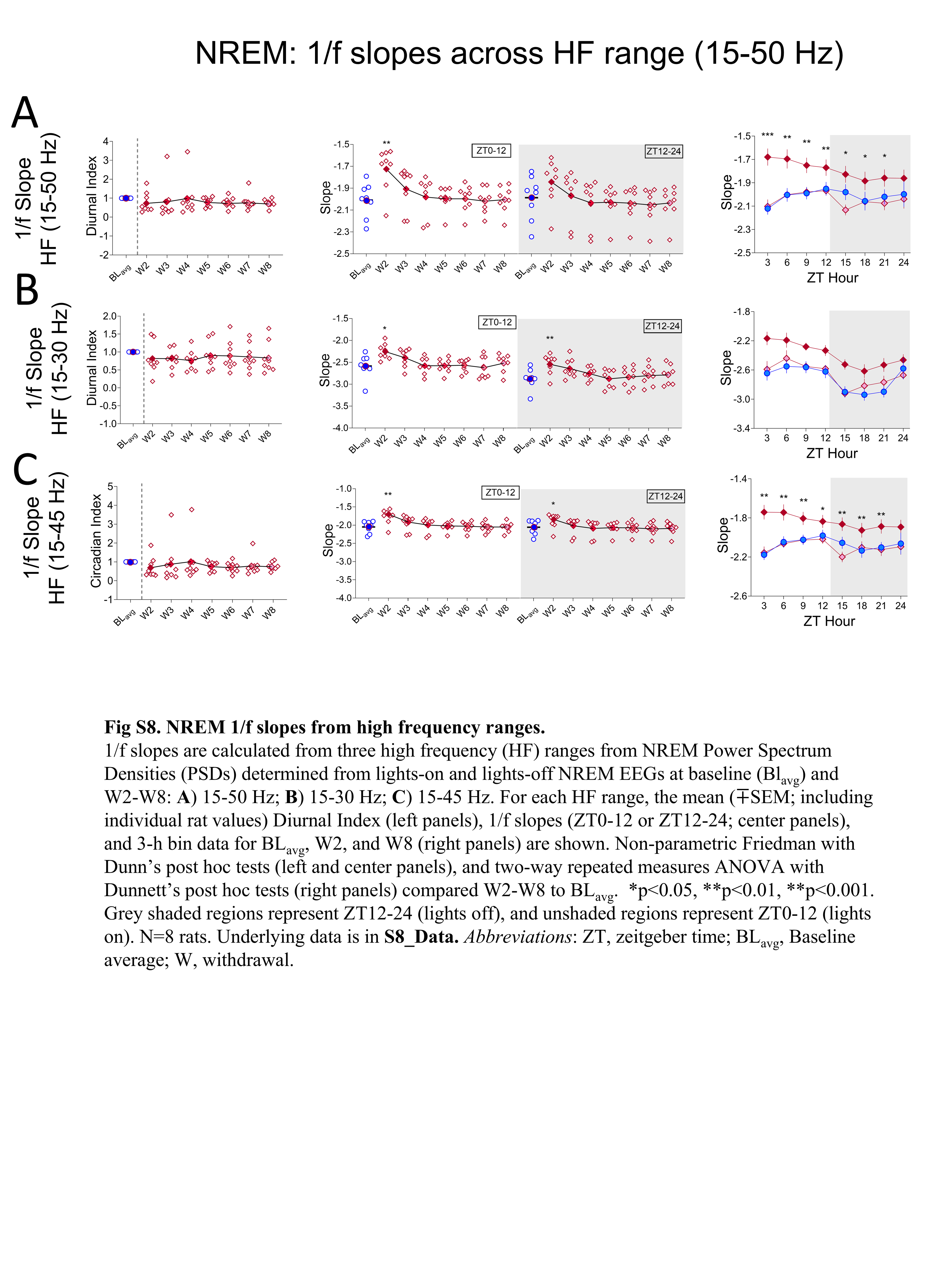

Supplement: S1 File — (ZIP) [file pone.0312794.s001.zip › All Supplementary Figures and Data 101824/S8_Fig.tif]

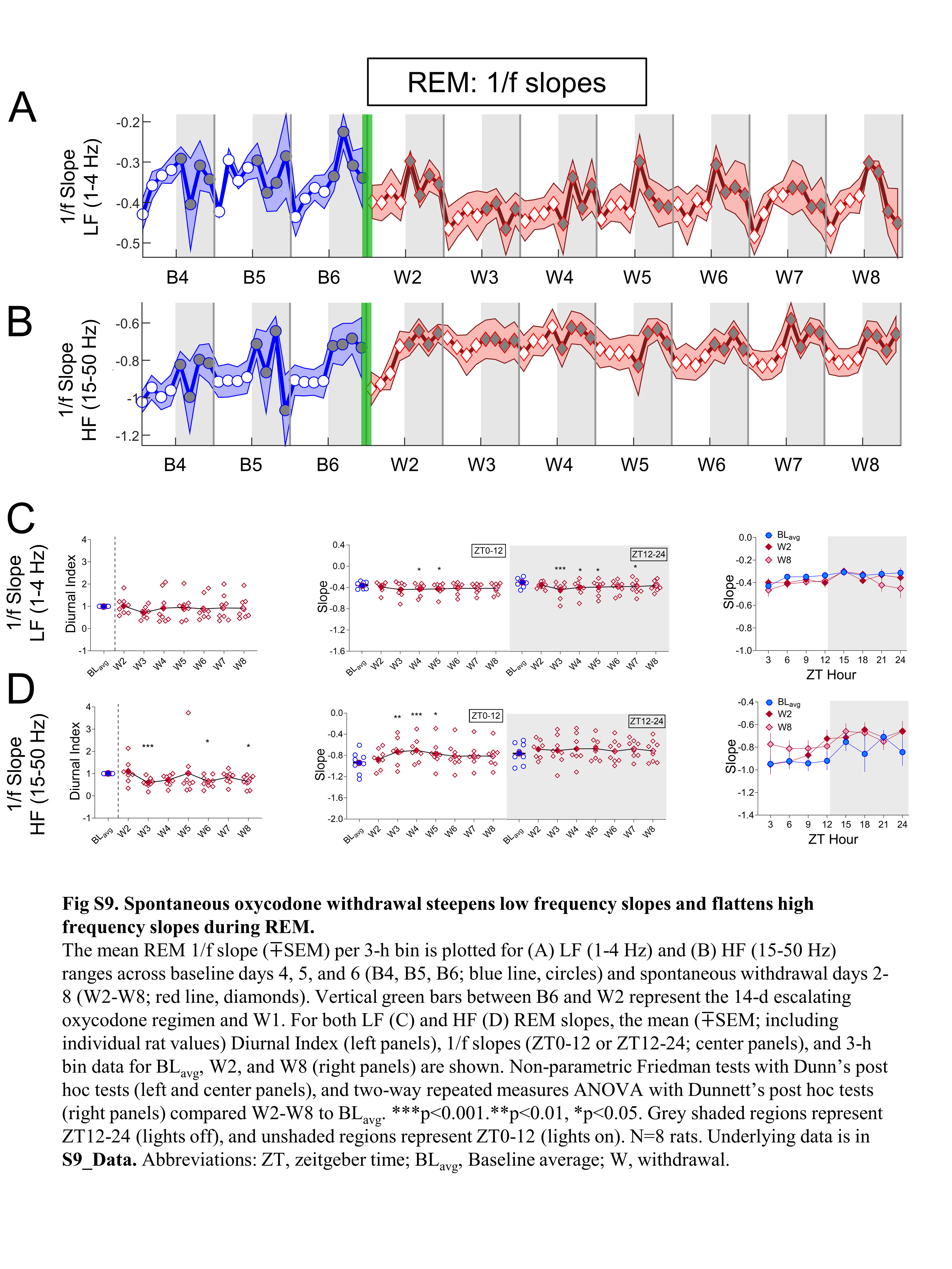

Supplement: S1 File — (ZIP) [file pone.0312794.s001.zip › All Supplementary Figures and Data 101824/S9_Fig.tif]
